# The occurrence of potato common scab correlates with the community composition and function of the geocaulosphere soil microbiome

**DOI:** 10.1186/s40168-019-0629-2

**Published:** 2019-02-01

**Authors:** Wencong Shi, Mingcong Li, Guangshan Wei, Renmao Tian, Cuiping Li, Bing Wang, Rongshan Lin, Chunyu Shi, Xiuli Chi, Bo Zhou, Zheng Gao

**Affiliations:** 1 State Key Laboratory of Crop Biology, Shandong Agricultural University, Tai’an, 271018 China; 2 College of Life Sciences, Shandong Agricultural University, Tai’an, 271018 China; 3 Key Laboratory of Marine Genetic Resources, Third Institute of Oceanography, SOA, Xiamen, 361005 China; 4 South China Sea Resource Exploitation and Protection Collaborative Innovation Center (SCS-REPIC), Sun Yat-Sen University, Guangzhou, 510275 China; 5 Department of Botany and Microbiology, Institute for Environmental Genomics, University of Oklahoma, Norman, USA; 6 National Engineering Laboratory for Efficient Utilization of Soil and Fertilizer Resources, Tai’an, 271018 China; 7 College of Agronomy, Shandong Agricultural University, Tai’an, 271018 China; 8 Plant Protection Station, Jiaozhou Agricultural Bureau, Qingdao, 266300 China

**Keywords:** Soil microbiome, Common scab, Geocaulosphere soil, Metagenome, Microbial community composition and function

## Abstract

**Background:**

Soil microorganisms can mediate the occurrence of plant diseases. Potato common scab (CS) is a refractory disease caused by pathogenic *Streptomyces* that occurs worldwide, but little is known about the interactions between CS and the soil microbiome. In this study, four soil-root system compartments (geocaulosphere soil (GS), rhizosphere soil (RS), root-zone soil (ZS), and furrow soil (FS)) were analyzed for potato plants with naturally high (H) and low (L) scab severity levels. We aimed to determine the composition and putative function of the soil microbiome associated with potato CS.

**Results:**

The copy numbers of the scab phytotoxin biosynthetic gene *txtAB* and the bacterial 16S rRNA gene as well as the diversity and composition of each of the four soil-root system compartments were examined; GS was the only compartment that exhibited significant differences between the H and L groups. Compared to the H group, the L group exhibited a lower *txtAB* gene copy number, lower bacterial 16S copy number, higher diversity, higher co-occurrence network complexity, and higher community function similarity within the GS microbiome. The community composition and function of the GS samples were further revealed by shotgun metagenomic sequencing. *Variovorax*, *Stenotrophomonas*, and *Agrobacterium* were the most abundant genera that were significantly and positively correlated with the scab severity level, estimated absolute abundance (EAA) of pathogenic *Streptomyces*, and *txtAB* gene copy number. In contrast, *Geobacillus*, *Curtobacterium*, and unclassified *Geodermatophilaceae* were significantly negatively correlated with these three parameters. Compared to the function profiles in the L group, several genes involved in “ABC transporters,” the “bacterial secretion system,” “quorum sensing (QS),” “nitrogen metabolism,” and some metabolism by cytochrome P450 were enriched in the H group. In contrast, some antibiotic biosynthesis pathways were enriched in the L group. Based on the differences in community composition and function, a simple model was proposed to explain the putative relationships between the soil microbiome and CS occurrence.

**Conclusions:**

The GS microbiome was closely associated with CS severity in the soil-root system, and the occurrence of CS was accompanied by changes in community composition and function. The differential functions provide new clues to elucidate the mechanism underlying the interaction between CS occurrence and the soil microbiome, and varying community compositions provide novel insights into CS occurrence.

**Electronic supplementary material:**

The online version of this article (10.1186/s40168-019-0629-2) contains supplementary material, which is available to authorized users.

## Background

Soil microorganisms, which closely interact with plants, are an important factor affecting plant health [1–3]. The emergence and propagation of plant pathogens cause plant diseases, and in turn, soil microorganisms can prevent plant diseases and inhibit pathogens to maintain plant health [4, 5]. Using microorganisms to control plant diseases has been successful in many ways, and biocontrol is considered a desirable approach for controlling soil-borne diseases [6–9]. Many biocontrol agents for plant pathogens have been developed and applied, but due to the complexity of the soil environment and of soil microbial interactions, the effectiveness of biocontrol agents often differs under laboratory and natural environment conditions. To ensure that the biocontrol of plant disease is more stable and more effective in the natural environment, a clear understanding of how pathogens, biocontrol strains, and soil microorganisms interact is necessary. Therefore, it is of great significance to study the soil microbial community to control plant diseases.

In the last decade, an increasing number of studies have focused on the role of the soil microbial community in the field of plant disease prevention and treatment [3, 10–12]. Disease occurrence is often accompanied by changes in the microbial community, such as altered microbial abundance, composition, and function [13, 14], so additional efforts to rehabilitate the microbial community may be more effective at curing plant diseases than simply controlling pathogen populations. Research indicates that the diversity of soil microbial communities is closely related to plant disease resistance, and high microbial diversity provides plants with greater protection from invasion by pathogens [15–17]. The increased pathogen invasion resistance of soil with high microbial diversity is likely related to the complexity of the microbial interaction network in the soil [18–20]. Complex microbial community interactions can regulate community stability [20–22], thereby controlling pathogen propagation. Therefore, studying the interaction between plant pathogens and the soil microbiome is necessary for more effective disease prevention.

Potato common scab (CS) is an economically important disease that occurs worldwide and alone was estimated to have caused approximately 15.3–17.3 million Canadian dollars in economic losses in Canada in 2002 [23]. Previous studies have suggested that CS is independently caused by several different *Streptomyces* species, including *S*. *scabies* [24], *S*. *acidiscabies* [25], *S*. *turgidiscabies* [26], *S*. *europaeiscabiei* [27], *S*. *stelliscabiei* [27], and *S*. *bottropensis* [28]. Pathogenic *Streptomyces* spp. live not only on the skin of potato tubers as a parasite but also in the soil as a saprophyte. This pathogen produces a family of phytotoxins called thaxtomins that are cyclic dipeptides (2,5-diketopiperazines) containing 4-nitrotryptophan and phenylalanine residues [29, 30], and thaxtomin A (ThxA), the most prominent member of the family, has been confirmed to be associated with *Streptomyces* pathogenicity [31–33]. ThxA biosynthetic genes reside on a mobile pathogenicity island (PAI) in pathogenic *Streptomyces* genomes [34–36]. The biosynthetic pathway of ThxA involves two nonribosomal peptide synthetases (NRPS) encoded by *txtA* and *txtB* genes, a P450 monooxygenase (TxtC), a nitric oxide synthase (TxtD), and a novel cytochrome P450 (TxtE) [30, 37–40]. ThxA on plant cells has been shown to inhibit cellulose biosynthesis, promote plant cell hypertrophy, and cause programmed cell death [41–43].

How to control CS has long been a thorny problem and has attracted global attention. To date, many practices have been used to control CS that are often not effective [44–46]. Many studies have focused on the treatment of CS through biocontrol agents, and some microorganisms, such as some strains of *Bacillus* [47, 48] and *Pseudomonas* [49, 50] and some non-pathogenic *Streptomyces* species [51–53], have been proven to reduce the incidence and/or severity of CS. However, there is still a lack of systematic, in-depth research on the soil microbiome of CS, and its interactions with soil microorganisms remain largely unclear. In this study, we investigated the soil microbiome associated with different scab severities (H: high scab severity; L: low scab severity) of potato plants. Four soil-root system compartments, geocaulosphere (tuber surface) soil (GS), rhizosphere soil (RS), root-zone soil (ZS), and furrow soil (FS), were collected from around each plant and analyzed. A series of approaches, including quantitative PCR (qPCR) and bacterial 16S rRNA gene amplicon sequencing, were implemented to explore the microbiomes of all soil samples, and additional shotgun metagenomic sequencing was applied to GS to explore microbial function. We specifically sought to (i) determine the range of the soil that its microbial community characteristics are closely associated with CS, (ii) clarify the composition and functional profiles of the soil microbiome in L plants, and (iii) explain the interactions between the soil microbial community and CS occurrence.

## Materials and methods

### Field experiment

The field experiment was conducted in 2015. All potatoes were planted in Anjiatun Village (34.248727° N, 119.816724° E, 22.9 m a.s.l.) of Jiaozhou City in Shandong Province, China on August 15th. The field was approximately 50-m long and 7-m wide and had nine ridges parallel to the long side (Additional file 1: Figure S1) that were 20–30 cm high, and two adjacent ridges were spaced 70–80 cm apart. The potatoes were planted on the ridges in a single row with a spacing of approximately 20–25 cm between plants. Favorita 15, the potato cultivar studied here, is susceptible to CS. The sampled field had a potato planting history of many years, and CS had occurred during the last planting.

### Sampling

Samples were collected on November 3, 2015 (80 days after planting), and the four soil-root system compartments of each plant including GS, RS, ZS, and FS were selected to fully explore the relationship between the soil microbiome and CS severity in the soil-root system. Plant population density, appearance, growth rate, and growth period were considered in our analysis; plants with an unusual size, pests, or mechanical damage as well as those impacted by marginal effects were excluded. The tubers and roots were carefully collected with an aseptic stainless-steel shovel, and the soil that was loosely attached to the tubers and roots was gently removed. CS severity was evaluated using a scale of 1 to 9 based on the percentage of the surface covered by lesions: 1: no scab; 2: 0.1–0.8%; 3: 0.9–2.8%; 4: 2.9–7.9%; 5: 8.0–18.0%; 6: 18.1–34.0%; 7: 34.1–55.0%; 8: 55.1–77.0%; and 9: 77.1–100% [54]; the coverage range was evaluated based on simple measurements with a grid ruler. A total of ten plants were selected according to scab severity. Plants No. 1 to 5 were grouped and identified as H (scab severity rank ≥ 4 for each tuber), and No. 6 to 10 were grouped and identified as L (scab severity rank 1–2 for each tuber). To accurately measure the relationship between the soil microbiome and scab severity, one tuber per plant was chosen, and GS and RS were collected as the soil tightly attached to the tuber and root surfaces, respectively, as follows [55]. Briefly, the tuber or root was stirred vigorously in a sterile phosphate-buffered saline (PBS) solution to wash all the soil from the surface, and the soil was then collected by high-speed centrifugation. ZS was collected from the region of root growth under the plant, and FS was collected at a depth of 5–10 cm from the furrow next to the plant (FS1 was missing). All samples were stored at low temperature in ice bags (~ 4 °C) and transported to the laboratory within 12 h. After homogenization, a portion of each sample was stored at − 80 °C until DNA extraction, and the remainder of the sample was immediately processed for physicochemical analysis.

### Isolation and identification of *Streptomyces* strains

A pure culture experiment was performed to isolate *Streptomyces* strains from potato lesions using the enrichment culture law and dilution on agar-medium plates. The lesions were homogenized, combined with 9 mL sterile water, incubated at room temperature for 30 min, diluted 10 times and 100 times with sterile water, and then coated on oatmeal agar plates (OMA; 10 g oatmeal, 18 g Bacto agar, and nutrients: 1 g MgSO_4_·7H_2_O, 1.5 g KH_2_PO_4_, and 1 g NaNO_3_ per liter) for culture at 28 °C for 3–7 days. Single colony-forming units were selected and sub-cultured three times on OMA plates, and the 16S rRNA genes of the isolated strains were amplified with the bacterial universal primers 27F (5′-GAGAGTTTGATCCTGGCTCAG-3′) and 1492R (5′-ACGGATACCTTGTTACGACT-3′). PCR amplicons were verified on a 1% agarose gel, and the reaction products were purified and sequenced on an ABI 3730XL DNA Analyzer (Applied Biosystems, USA). The 16S sequences were aligned with an NCBI 16S ribosomal RNA sequences (Bacteria and Archaea) database by Nucleotide BLAST (https://blast.ncbi.nlm.nih.gov/Blast.cgi) to determine the approximate phylogenetic affiliation of the strains. Taxonomy was confirmed if the maximum identity of the sequence reported by NCBI BLAST was > 97% and was the greatest of all listed matches. A phylogenetic tree was constructed to visualize the phylogenetic relationships using the neighbor-joining method by MEGA7 software.

### Physicochemical analysis

The physicochemical characteristics of ZS and FS were assayed in three technical replicates to evaluate the soil conditions in the study field. Soil pH was measured using a mixture of soil and deionized water free of CO_2_ at a ratio of 1:2.5 (*w*/*v*). Soil total carbon (TC), organic matter (OM), total nitrogen (TN), ammonium (NH_4_^+^-N), nitrate (NO_3_^−^-N), available phosphorus (AP), available potassium (AK), and available sulfur (AS) were determined following previous studies [56, 57].

### DNA extraction

GS, RS, ZS, and FS DNA were extracted using the E.N.Z.A.™ Soil DNA Kit (Omega, USA) according to the manufacturer’s instructions, and DNA quantity and quality were determined using a NanoDrop 2000 spectrophotometer (Thermo Scientific, USA). DNA was stored at − 80 °C until further analysis.

### Quantitative PCR

The bacterial 16S rRNA gene and thaxtomin biosynthetic gene *txtAB* copy numbers were detected by qPCR in thre technical replicates. The primers 515F (5′-GGACTACVSGGGTATCTAAT-3′) and 806R (5′-GTGCCAGCMGCCGCGGTAA-3′) were used to amplify a 292-bp fragment of the 16S rRNA gene from bacteria [58], and the primers StrepF (5′-GCAGGACGCTCACCAGGTAGT-3′) and StrepR (5′-ACTTCGACACCGTTGTCCTCAA-3′) were used to amplify a 72-bp fragment of the thaxtomin biosynthetic gene *txtAB* from scab-pathogenic *Streptomyces* [59]. The analyses were performed on a CFX96™ real-time system (Bio-Rad, USA) using 96-well plates. Amplifications were performed in a final volume of 20 μL containing 10 μL of SYBR® Premix Ex Taq™ (Tli RNaseH Plus; Takara, China), 5 μL of DNA, 0.2 μL of each 10-μM primer, and 4.6 μL of ultra-pure water. Plasmids containing either the 16S rRNA gene fragment or *txtAB* gene fragment were constructed to prepare the respective standard curves, and the plasmid copy numbers were automatically calculated using an online calculator (cels.uri.edu/gsc/cndna.html) based on the concentration, size, and average weight of a base pair. Standard curve equations that showed the relationship between gene copy numbers and Ct values were generated with serial dilutions of the above plasmids with known copy numbers after real-time fluorescence qPCR. After the same PCR runs, the copy numbers of samples were calculated based on the standard curve equations and their own Ct values. No-template controls as well as positive controls with known Ct values were included in every PCR. The thermocycling conditions for amplification of both the bacterial 16S and *txtAB* genes were 95 °C for 5 min; 40 cycles of 95 °C for 10 s, 55 °C for 10 s, and 72 °C for 20 s; and a final melting cycle to produce a melting curve for quality control. All runs had standard efficiency curves of *r*^2^ > 0.99 and efficiencies of 90–110%.

### Illumina MiSeq sequencing and analysis

The primers 341F (5′-CCTACGGGNGGCWGCAG-3′) and 805R (5′-GACTACHVGGGTATCTAATCC-3′) were used to amplify the V3-V4 hypervariable region of the 16S rRNA gene. Amplicon libraries were constructed using the NEB Next® Ultra™ DNA Library Prep Kit for Illumina (NEB, USA) following the manufacturer’s recommendations, and index codes were added. The amplicon libraries were sequenced on a MiSeq PE250 sequencer (Illumina, USA), and 250-bp paired-end reads were generated. Approximately, 2.2 GB raw reads were generated after Illumina sequencing, and the resulting paired sequence reads were then merged, trimmed, filtered, aligned, and clustered by operational taxonomic unit (OTU) using USEARCH v. 9.2 software [60]. Once the paired sequence reads were merged, a total of 1,878,784 merged sequences were generated, with an average of 48,174 sequences for each soil sample (minimum = 43,903; maximum = 51,875). Sequences with ≥ 97% similarity were assigned to the same OTU by the UPARSE-OTU algorithm in USEARCH, and chimeras were filtered during OTU clustering using the cluster_otus command. Sequences of plastids and mitochondria and those not classified in the domain Bacteria were discarded as were OTUs with a sequence number of 1 in all samples. Taxonomy assignment was performed using the USEARCH-recommended database: the RDP training set for 16S. Alpha- and beta-diversity indices were calculated based on the rarefied OTU table at a depth of 29,718 sequences per sample. Alpha-diversity indices, including the ACE, Chao1, coverage, Shannon, and Simpson indices, were calculated with Mothur v. 1.34.4 [61]. Command lines for the above data processing are available in Additional file 2.

### Shotgun metagenomic sequencing and analysis

Based on the results of the above 16S amplicon sequencing, GS DNA was selected for shotgun metagenomic sequencing to evaluate the microbial community composition and function with higher resolution. Metagenomic libraries were constructed using a TruSeq™ DNA PCR-free Sample Prep Kit (Illumina, USA) according to the manufacturer’s instructions. The metagenomic libraries were sequenced on a HiSeq 2500 sequencer (Illumina, USA), and 150-bp paired-end reads were generated. Approximately 244.4 GB raw reads were generated after Illumina sequencing, with a total of 727,482,738 resulting paired sequence reads for all 10 GS samples.

The resulting sequence reads were analyzed for quality control using a range of software programs: SeqPrep (https://github.com/jstjohn/SeqPrep) for tripping non-biological bases in reads, such as primers or barcodes, and Sickle (https://github.com/najoshi/sickle) for filtering reads whose length after tripping was less than 50 bp and whose average quality score was less than 20. Then, 52.4–80.5 million clean reads per sample were obtained. The optimized sequence reads were assembled de novo by SOAPdenovo (http://soap.genomics.org.cn/, Version 1.06) based on a de Bruijn graph for obtaining contigs, and a total of 1,088,639 contigs were generated. Resulting contigs > 500 bp in length were selected to predict open reading frames (ORFs) using MetaGene (http://metagene.cb.k.u-tokyo.ac.jp/), and 1,731,093 ORFs were obtained. All predicted genes were aligned pairwise using CD-HIT (http://www.bioinformatics.org/cd-hit/), and those for which more than 90% of their length could be aligned to another gene with more than 95% identity (no gaps allowed) were removed as redundancies excepted for the longest gene, resulting in a non-redundant gene catalog comprised of 1,205,798 genes with an average length of 579.02 bp.

#### Computation of gene relative abundance

The high-quality reads from each sample were aligned against the gene catalog by SOAPaligner (http://soap.genomics.org.cn/) using the criterion “identity > 95%.” In each sample, the mapped reads of each gene were counted as the number of gene-mapped reads, and the gene relative abundance was calculated following a previous study [62].

$$ {a}_i=\frac{b_i}{\sum_j{b}_j}=\frac{\frac{x_i}{L_i}}{\sum_j\frac{x_j}{L_j}} $$

*a*_*i*_: The relative abundance of gene *i* in sample *S*.

*L*_*i*_: The length of gene *i*.

*x*_*i*_: The times at which gene *i* could be detected in sample *S* (the number of mapped reads).

*b*_*i*_: The copy number of gene *i* in the sequenced data from sample *S*.

#### Functional annotation

We aligned putative amino acid sequences, which were translated from the gene catalog, against the proteins/domains in KEGG databases (Release 79.0) using BLASTP (BLAST v. 2.2.28+, http://blast.ncbi.nlm.nih.gov/Blast.cgi) (*e* value ≤ 1e-5). A total of 776,122 genes were hit in the KEGG databases and were assigned to 3328 KEGG orthology (KO) functional categories and 365 KEGG pathways.

#### Taxonomic assignment of genes

We aligned putative amino acid sequences translated from the gene catalog against the proteins/domains in the NCBI-NR database (downloaded 01/2017) using BLASTP (BLAST v. 2.2.28+, http://blast.ncbi.nlm.nih.gov/Blast.cgi) (*e* value ≤ 1e-5). Genes were taxonomically annotated using corresponding taxonomic information from the NR database. In each sample, the mapped reads of each taxon were counted as the number of taxon-mapped reads.

#### Taxonomic profiling and abundance statistics

We profiled the composition of microbial communities (Bacteria, Archaea, Eukaryotes and Viruses) from raw shotgun metagenomic sequencing reads using MetaPhlAn v. 2.0 [63], and the bacterial taxonomic profiling was subsequently separated from the microbial taxonomic profiling. The relative abundances of microbial taxonomic profiling were quantified by unique clade-specific marker genes of the MetaPhlAn v. 2.0 reference database and then normalized to values in proportion. The estimated absolute abundance (EAA) of bacterial taxa was calculated using the method described by Zhang [64].

*EAA*_*i*_ = *a*_*i*_ × *q*_*s*_*.*

*EAA*_*i*_: The estimated absolute abundance of bacterial taxon *i* in sample *S*.

*a*_*i*_: The relative abundance of bacterial taxon *i* in sample *S*.

*q*_*s*_: The copy number of bacteria from sample *S* detected by qPCR.

### Statistical analysis

The two-tailed Wilcoxon rank-sum test was performed using the base R package “stats” (v. 3.4.1) wilcox.test function. Spearman’s correlation coefficient and significance were calculated using the rcorr function in the R package “Hmisc” (v. 4.0-3). The Bray-Curtis metric was calculated using the vegdist function from the R package “vegan” (v. 2.4-4). Principal coordinates analysis (PCoA) was performed using the prcomp function from the R package “stats” with the Bray-Curtis metric. Analysis of similarities (ANOSIM) was performed with the anosim function from the R package “vegan” with the Bray-Curtis metric. KEGG pathway enrichment analysis was performed using the function phyper from the R package “stats”. The linear discriminant analysis (LDA) effect size (LEfSe) statistical analysis was performed on the online interface Galaxy (http://huttenhower.sph.harvard.edu/lefse/) with an alpha value < 0.05 and an LDA score > 2. The correlation network was visualized using Cytoscape v. 3.6.0.

## Results

### The low scab severity group was characterized by a GS microbiome with low *txtAB* gene abundance, low bacterial abundance, and high diversity

The physicochemical characteristics of ZS and FS were assayed to evaluate the soil conditions of the study field, and the results are described in Additional file 1: Table S1. The physicochemical analyses in the present study indicated that CS occurred in acidic soil conditions, and the pH values of ZS and FS ranged from 4.32 to 6.81. A two-tailed Wilcoxon rank-sum test was performed to identify differences between the physicochemical characteristics in H and L (Additional file 1: Table S1). The concentration of ammonium (NH_4_^+^-N) in ZS was significantly different (two-tailed Wilcoxon test, *P* = 0.03) between the H and L groups, but there was no significant difference in all observed physicochemical characteristics in FS. In addition to ammonium (two-tailed Wilcoxon test, *P* = 0.01), the concentrations of soil TC (two-tailed Wilcoxon test, *P* = 0.02) and OM (two-tailed Wilcoxon test, *P* = 0.01) were also significantly different between the H and L groups when combining ZS and FS.

**Fig. 1:**
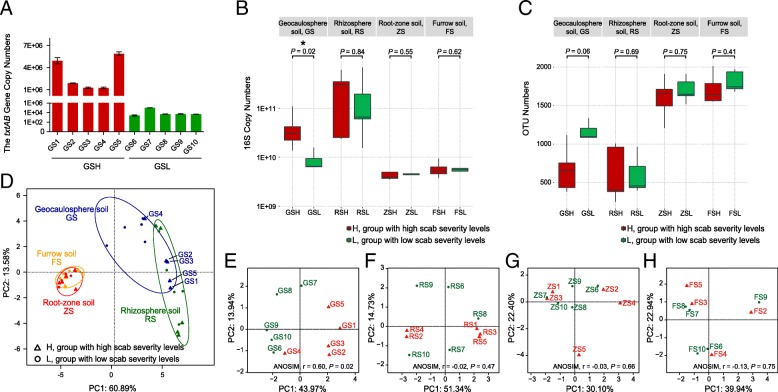
CS separated the bacterial community of GS but not that of RS, ZS, or FS. **a** The copy number of the thaxtomin biosynthetic gene, *txtAB*, was significantly higher for GS in the H group than in the L group. Differences between high and low scab severity levels were observed in **b** bacterial 16S copy numbers, **c** OTU numbers, and **d**–**h** the principal coordinates analysis (PCoA) of bacterial community compositions in GS, but no significant differences were observed in RS, ZS, or FS. The error bars indicate the SD of three replicates, and asterisks indicate statistical significance (two-tailed Wilcoxon test, *P < 0.05). The dissimilarity of bacterial compositions at the OTU level was visualized by PCoA based on the Bray-Curtis metric. Triangles denote samples in group H (GSH, RSH, ZSH, and FSH; No. 1 to No. 5; high scab severity levels), and circles denote samples in group L (GSL, RSL, ZSL, and FSL; No. 6 to No. 10; low scab severity levels). Group H is shown colored in red, and group L is shown in green (**a**–**h**, except **d**). In (**d**), GS, RS, ZS, and FS are shown in blue, green, red, and orange, respectively

*TxtAB* is the key gene for the biosynthesis of scab phytotoxin ThxA and enables the accurate quantification of pathogenic *Streptomyces* strains [65]. To determine scab pathogen abundance, all soil samples were used to detect the copy numbers of the thaxtomin biosynthetic gene *txtAB*, but only GS produced clear fluorescent signals in the real-time PCR system (Fig. 1a), indicating that GS contained a high abundance of the scab pathogen while the other samples contained little or no scab pathogen. Furthermore, the gene *txtAB* copy numbers of the scab pathogen were significantly (two-tailed Wilcoxon rank-sum test, *P* = 0.008) different between GSH and GSL and were significantly positively correlated with scab severity level (Spearman, ρ = 0.97, *P* = 5.55 × 10^−6^).

The bacterial 16S rRNA gene copy numbers were detected by qPCR to reveal the distribution of bacterial abundance in all soil samples. The highest bacterial copy numbers were detected in RS (compared to GS; two-tailed Wilcoxon test, *P* = 0.004), ranging from 1.55 × 10^10^ ± 8.06 × 10^8^ to 6.82 × 10^11^ ± 3.10 × 10^10^ copies/g soil, followed by GS, ranging from 1.29 × 10^9^ ± 9.62 × 10^7^ to 1.08 × 10^11^ ± 5.17 × 10^9^; interestingly, only GS had significantly (two-tailed Wilcoxon test, *P* = 0.02) different bacterial copy numbers between GSH and GSL (Fig. 1b). The mean bacterial copy number in GSH was 4.33 × 10^10^, and the mean in GSL was 7.84 × 10^9^. The copy numbers for GS were significantly positively correlated with scab severity level (Spearman, ρ = 0.80, *P* = 0.005), indicating that increased bacterial abundance in GS may signal the occurrence of CS.

The 16S rRNA genes of all soil samples were sequenced on a MiSeq PE250 sequencer, and all statistical calculations were performed based on rarefied OTU profiling at a depth of 29,718 sequences per sample (Additional file 3). A total of 2700 OTUs were yielded from the bacterial community. The number of OTUs in each soil compartment is shown in Fig. 1c for comparison between the H and L groups, but the statistical significance (*P* value < 0.05) was not evaluated in any soil compartment by two-tailed Wilcoxon test (GS: *P* = 0.06; RS: *P* = 0.69; ZS: *P* = 0.75; FS: *P* = 0.41). Compared with the other compartments, the difference in GS was nearly statistically significant, with a *P* value = 0.06, and showed a slightly lower OTU number in GSH than in GSL (Fig. 1c). Notably, a left-tailed Wilcoxon test showed a significant difference (*P* = 0.03) between GSH and GSL. Significant differences were also evaluated by *t* test (two-tailed *t* test, *P* = 0.03; left-tailed *t* test, *P* = 0.02), but none of the other soil compartments were tested for significant differences using the same methods (two-tailed *t* test, left-tailed Wilcoxon test, left-tailed *t* test, *P* > 0.05). The bacterial OTU numbers of GS were significantly negatively correlated with scab severity level (Spearman, ρ = − 0.83, *P* = 0.003). Furthermore, alpha-diversity indices (Additional file 1: Table S2) were calculated with Mothur v. 1.34.4 [61], and the results showed that the Chao1 index (two-tailed Wilcoxon test, *P* = 0.03; left-tailed Wilcoxon test, *P* = 0.02; left-tailed *t* test, *P* = 0.01) and Shannon index (two-tailed Wilcoxon test, *P* = 0.15; left-tailed Wilcoxon test, *P* = 0.08; left-tailed *t* test, *P* = 0.03) approached marginally significant differences between GSH and GSL.

### Significant differences in bacterial community composition were observed in GS between high and low scab severity levels

To visualize the similarity and dissimilarity in bacterial communities among soil samples, PCoA was performed based on bacterial OTUs of 16S rRNA gene amplicon sequencing using the Bray-Curtis metric. All samples were primarily clustered by soil compartment type (ANOSIM, *r* = 0.5461, *P* = 0.001) rather than by scab severity (ANOSIM, *r* = 0.009, *P* = 0.26) (Fig. 1d). Within the same soil-root system compartment, bacterial communities in GS could be distinguished based on CS (Fig. 1e; ANOSIM, *r* = 0.60, *P* = 0.02), but this was not the case for RS (Fig. 1f; ANOSIM, *r* = − 0.02, *P* = 0.47), ZS (Fig. 1g; ANOSIM, *r* = − 0.03, *P* = 0.66), or FS (Fig. 1h; ANOSIM, *r* = − 0.13, *P* = 0.75). This finding indicates that there were significant differences in the GSH and GSL bacterial community compositions. ZS and FS highly overlapped, indicating that the bacterial communities of these two soil compartments were generally similar, but they still had a few different characteristics (ZS vs. FS; ANOSIM, *r* = 0.24, *P* = 0.003).

The two-tailed Wilcoxon rank-sum test was used to evaluate differences in bacterial relative abundance at the genus level among groups (Table 1). Consistent with the PCoA results, the largest number of differentiated (*P* < 0.05) genera existed between different soil compartments. A total of 143 differentiated bacterial genera were found between RSL and FSL, and 132 were found between GSH and FSH. Notably, 53 differentiated bacterial genera were found between GSL and RSL, while only 7 differentiated bacterial genera were found between GSH and RSH, indicating that H might reduce the community differences between GS and RS. Of the four soil-root system compartments, GS contained 58 differentiated (*P* < 0.05) bacterial genera between H and L, which was far greater than the 1, 5, and 7 identified in RS, ZS, and FS, respectively. This finding further demonstrates that the bacterial community composition of GS was related to CS severity.

**Table 1:** Numbers of significantly (*P* < 0.05) differentiated bacterial genera between groups evaluated by two-tailed Wilcoxon test

	GSH	RSH	ZSH	FSH	GSL	RSL	ZSL	FSL
GSH		7	106	132	58			
RSH			104	120		1		
ZSH				25			5	
FSH								7
GSL						53	91	102
RSL							130	143
ZSL								42
FSL								

The LEfSe method was applied to identify differences in taxonomic abundance between GSH and GSL (Additional file 1: Figure S2 and S3). The taxa enriched in GSH were mainly found in *Proteobacteria* and *Bacteroidetes*, and the taxa enriched in GSL mainly belonged to *Acidobacteria*, *Actinobacteria*, and *Firmicutes*. At the genus level, 67 genera were found to be significantly (*P* < 0.05) different between GSH and GSL, of which 12 genera were enriched in GSH and 55 were enriched in GSL. All differentiated genera identified by the Wilcoxon test and LEfSe analysis are depicted in Additional file 1: Figure S3. Among them, *Sphingomonas* (significantly enriched in GSL; Wilcoxon and LEfSe; *P* < 0.05), *Stenotrophomonas* (significantly enriched in GSH; LEfSe; *P* < 0.05), *Variovorax* (significantly enriched in GSH; Wilcoxon and LEfSe; *P* < 0.05), *Arthrobacter* (significantly enriched in GSL; Wilcoxon and LEfSe; *P* < 0.05), and *Sphingobium* (significantly enriched in GSH; Wilcoxon and LEfSe; *P* < 0.05) were abundant genera. Unexpectedly, the genus *Streptomyces* did not show a significant difference between GSH and GSL (two-tailed Wilcoxon test, *P* = 0.83).

### The low scab severity group was characterized by the GS microbiome with low pathogenic *Streptomyces* abundance

To obtain more information about the pathogens and microbial community function, GS DNA was selected for shotgun metagenomic sequencing via a HiSeq 2500 sequencer, after which a total of 657,254,147 high-quality sequence reads were obtained with approximately 52.4–80.5 million clean reads per sample. From those sequencing reads, a non-redundant gene catalog was obtained comprising 1,205,798 genes, of which 223,997 (45,621,568 clean reads) were taxonomically annotated by aligning them against the NR database (downloaded 01/2017). The sequence reads assigned to Bacteria (ca. 98.03%) occupied the dominant position, and a small proportion of sequence reads were assigned to Archaea (ca. 0.07%), Eukaryotes (ca. 1.44%), and Viruses (ca. 0.41%). According to the NR alignment results, 614,988 sequence reads belonged to the genus *Streptomyces*, and of these reads, 18 *Streptomyces* species represented by 243,560 sequence reads (0.53% of all reads, 39.60% of *Streptomyces* reads) were possible scab pathogens (Additional file 1: Table S4). Among these species, *S. acidiscabies* was dominant followed by *S*. *niveiscabiei*, *S*. *turgidiscabies*, and *S*. *scabiei*.

Relative abundances within the microbial community were quantified by MetaPhlAn v. 2.0 software (Additional file 4). Similar to the NR alignment results, the microbial community was composed of Bacteria (ca. 99.10%), Eukaryotes (ca. 0.02%), and Viruses (ca. 0.88%). As the main component of the microbial community, the bacteria were separated (Additional file 5), and two *Streptomyces* species in the metagenomic bacterial community, *S*. *acidiscabies* and *S*. *turgidiscabies*, were possible scab pathogens (Table 2). Similar to the NR alignment results, *S*. *acidiscabies* was the dominant scab pathogen analyzed by MetaPhlAn2 in our samples. In our culture experiment, 11 isolated strains had the highest phylogenetic similarity with the *S*. *acidiscabies* strain, and 1 had the highest similarity with the *S*. *turgidiscabies* strain (Additional file 1: Table S5; the sequences are in Additional file 6) according to the NCBI BLAST results; a phylogenetic tree constructed by MEGA7 software was used to visualize the phylogenetic relationships (Additional file 1: Figure S4). The culture experiment further confirmed the existence of *S*. *acidiscabies* and *S*. *turgidiscabies* and identified *S*. *acidiscabies* as the dominant pathogenic *Streptomyces* species in this study.

**Table 2:** Relative abundance of *Streptomyces* species in metagenomic bacterial taxonomic profiling (MetaPhlAn2)

Species	GS1	GS2	GS3	GS4	GS5	GS6	GS7	GS8	GS9	GS10	Pct in Bac	Pct in Strep
*Streptomyces acidiscabies*	4.82E-03	4.67E-03	2.32E-03	6.22E-03	1.44E-03	0.00E+ 00	4.09E-04	4.64E-04	1.71E-03	0.00E+00	0.2205%	69.18%
*Streptomyces turgidiscabies*	1.07E-03	1.01E-03	5.52E-04	1.32E-03	1.92E-04	4.80E-04	3.99E-04	0.00E+00	0.00E+00	0.00E+00	0.0502%	15.76%
*Streptomyces chartreusis*	0.00E+00	2.69E-04	5.85E-05	1.15E-04	0.00E+00	6.82E-04	1.03E-03	6.46E-04	5.52E-04	8.89E-05	0.0344%	10.81%
*Streptomyces flavogriseus*	0.00E+00	0.00E+00	4.65E-05	0.00E+00	0.00E+00	0.00E+00	9.57E-05	0.00E+00	7.24E-04	0.00E+00	0.0087%	2.72%
*Streptomyces lividans*	0.00E+00	0.00E+00	0.00E+00	0.00E+00	0.00E+00	0.00E+00	3.75E-04	0.00E+00	0.00E+00	7.23E-05	0.0045%	1.40%
*Streptomyces coelicolor*	0.00E+00	0.00E+00	0.00E+00	0.00E+00	0.00E+00	0.00E+00	4.30E-05	0.00E+00	0.00E+00	0.00E+00	0.0004%	0.14%
Sum	5.88E-03	5.95E-03	2.97E-03	7.65E-03	1.63E-03	1.16E-03	2.35E-03	1.11E-03	2.99E-03	1.61E-04	0.3187%	
Pathogenic *Streptomyces*	5.88E-03	5.69E-03	2.87E-03	7.54E-03	1.63E-03	4.80E-04	8.08E-04	4.64E-04	1.71E-03	0.00E+00	0.2707%	84.94%
Non-pathogenic *Streptomyces*	0.00E+00	2.69E-04	1.05E-04	1.15E-04	0.00E+00	6.82E-04	1.55E-03	6.46E-04	1.28E-03	1.61E-04	0.0480%	15.06%

Pct in Bac: the percentage of a taxon in bacteria; Pct in Strep: the percentage of a taxon in the genus *Streptomyces*

The sum of the relative abundances of *S*. *acidiscabies* and *S*. *turgidiscabies* was defined as the relative abundance of pathogenic *Streptomyces*; other *Streptomyces* species were non-pathogenic (Table 2). Consistent with the amplicon sequencing results (Additional file 1: Figure S3), the relative abundance of the entire *Streptomyces* genus was not significantly (two-tailed Wilcoxon test, *P* = 0.056) different between GSH and GSL, but interestingly, the relative abundance of pathogenic *Streptomyces* was significantly (two-tailed Wilcoxon test, *P* = 0.016) different. This result can be explained by the relative abundance of non-pathogenic *Streptomyces* being significantly (two-tailed Wilcoxon test, *P* = 0.021) enriched in GSL, suggesting that the relative abundance of the entire *Streptomyces* might not accurately reflect the distribution of the pathogenic taxa. The EAA of bacteria was calculated using the method described by Zhang [64], and that of pathogenic *Streptomyces* was revealed to be significantly (two-tailed Wilcoxon test, *P* = 0.008) enriched in GSH compared with GSL. The EAA of pathogenic *Streptomyces* exhibited extremely significant and positive correlations with the *txtAB* gene copy number (Spearman, ρ = 0.88, *P* = 0.0008) and scab severity level (Spearman, ρ = 0.89, *P* = 0.0005), but there were only moderately significant positive relationships between the relative abundance of pathogenic *Streptomyces* and *txtAB* gene copy number (Spearman, ρ = 0.68, *P* = 0.029) and scab severity level (Spearman, ρ = 0.66, *P* = 0.036). Overall, the abundance of pathogenic *Streptomyces* can reflect the scab severity level, and the EAA of pathogenic *Streptomyces* appears to be more suitable than relative abundance for reflecting *txtAB* gene copy number and scab severity level patterns (Additional file 1: Figure S5).

### The community composition and function of the GS microbiome were correlated with CS

**Fig. 2:**
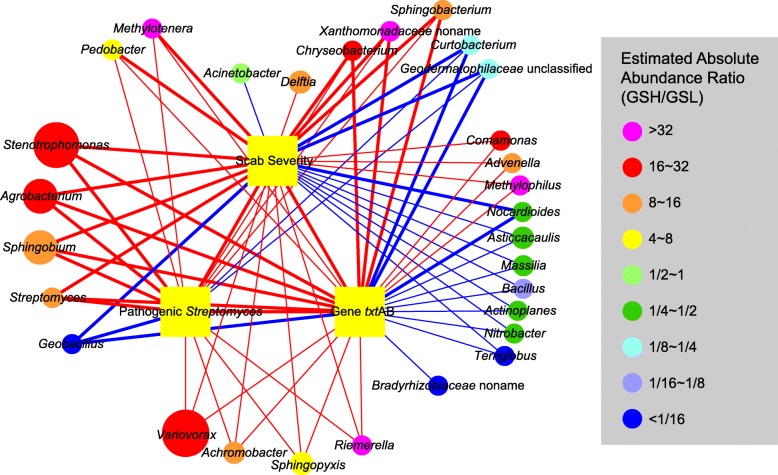
Interaction networks between the EAA of metagenomic bacteria (genus level) and scab severity level, the EAA of pathogenic *Streptomyces*, and the *txtAB* gene copy number. A connection represents a significant correlation (Spearman, |ρ| > 0.6, *P* < 0.05); thick lines indicate a Spearman’s correlation coefficient of |ρ| > 0.8; and thin lines indicate a Spearman’s correlation coefficient of 0.6 < |ρ| < 0.8. Red lines represent positive connections, while blue lines represent negative connections. The size of each node is proportional to the EAA of each genus. The color of each node represents the ratio of the EAA of each genus between GSH and GSL

According to the metagenomic data, the bacterial community was composed of 116 genera (Additional files 5 and 7). To clarify CS-sensitive genera in GS, three parameters, including the scab severity level, EAA of pathogenic *Streptomyces*, and *txtAB* gene copy number, were selected and analyzed using Spearman’s correlation analysis with the EAA of bacterial genera (Fig. 2). These three parameters were demonstrated to be significantly positively correlated with each other. Twenty-eight genera were significantly correlated (Spearman, |ρ| > 0.6, *P* < 0.05) with scab severity level, of which 17 were positive and 11 were negative. Sixteen genera were significantly correlated (Spearman, |ρ| > 0.6, *P* < 0.05) with the EAA of pathogenic *Streptomyces*, of which 13 were positive and 3 were negative. Twenty-seven genera were significantly correlated (Spearman, |ρ| > 0.6, *P* < 0.05) with *txtAB* gene copy number, of which 16 were positive and 11 were negative. Microorganisms associated with multiple parameters were considered to be of the utmost concern, and a total of 13 genera were significantly positively correlated (Spearman, |ρ| > 0.6, *P* < 0.05) with all 3 parameters. Notably, *Variovorax* exhibited the highest EAA of all associated genera; four genera (*Stenotrophomonas*, *Agrobacterium*, *Sphingobium*, and *Streptomyces*) exhibited a strong correlation (Spearman, ρ > 0.8, *P* < 0.05) with these three parameters. The significant correlation and high EAA ratios (GSH vs. GSL) raised the possibility that these genera were associated with CS. In addition, three genera (*Geobacillus*, *Curtobacterium*, and unclassified *Geodermatophilaceae*) were significantly negatively correlated (Spearman, − 1 ≤ ρ < − 0.6, *P* < 0.05) with all three parameters, and seven genera were significantly negatively correlated (Spearman, − 1 ≤ ρ < − 0.6, *P* < 0.05) with the scab severity level and the *txtAB* gene copy number but not with the EAA of pathogenic *Streptomyces*.

The co-occurrence network (Additional file 1: Figure S6) of the metagenomic bacterial community was used to compare the co-occurrence relationship and community complexity between GSH and GSL. Both the average number of neighbors and network density were higher in GSL, revealing a more complex co-occurrence relationship among these bacteria in GSL than in GSH. The network centralization values were 0.084 and 0.122 in GSH and GSL, respectively, and these low values indicated that the power of each microorganism was decentralized so that it was difficult for one or a few microorganisms to dominate and control the entire community. The occurrence of a higher network centralization value in GSL may be due to the existence of several large clusters with a higher degree of nodes, resulting in greater network centralization than that in GSH. A greater percentage of negative correlations were observed in GSH (27.91%) than in GSL (5.46%), implying that there may be greater antagonism among bacteria in GSH. Taken together, these results reveal a more complex co-occurrence community relationship in GSL, which might cause the GSL communities to be more resistant to pathogen invasion [18, 19].

**Fig. 3:**
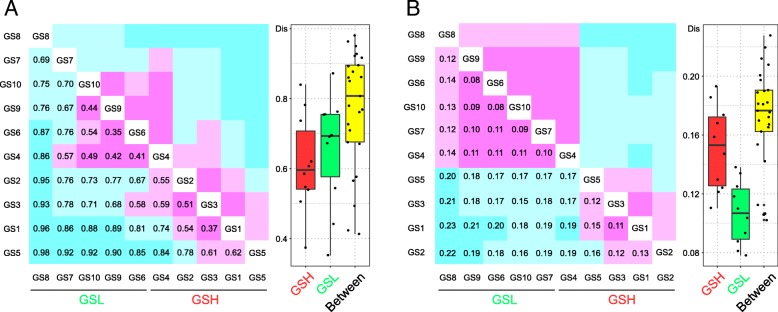
The dissimilarity metrics of **a** the EAA of metagenomic bacterial community composition (genus level) and **b** the relative abundance of KO functional categories based on Bray-Curtis dissimilarity. Magenta reflects a smaller dissimilarity coefficient (approach 0, the maximum similarity), and cyan represents a larger dissimilarity coefficient (approach 1, the minimum similarity) in the heatmap graph. Numbers in the graph indicate the dissimilarity coefficient. The box plot reveals the distribution of the dissimilarity coefficients within and between groups in the heatmap

The dissimilarity metrics based on Bray-Curtis ordination were calculated to quantify the compositional dissimilarity of both metagenomic bacterial community composition and KO functional category (Fig. 3). Our metagenomic functional profiling yielded a total of 3328 KO functional categories (Additional file 8), and in both microbial community composition and function, the dissimilarity between GSH and GSL was greater than that within groups (Within vs. Between; two-tailed Wilcoxon test; community composition, *P* = 6.3 × 10^−13^; community function, *P* = 0.0005), indicating that both community composition and function differed between GSH and GSL. The dissimilarity coefficient values for community function were lower than those for community composition (two-tailed Wilcoxon test, *P* < 2.2 × 10^−16^), demonstrating that the microbial function of GS was more similar than its community composition. In particular, the community function of GSL exhibited a significantly (two-tailed Wilcoxon test, *P* = 0.003) lower dissimilarity than that of GSH, whereas the bacterial community composition of GSL showed a slightly higher (two-tailed Wilcoxon test, *P* = 0.53) dissimilarity. In other words, the microbial functional profiling of GSL was more similar than that of GSH, although the dissimilarity of its community composition was slightly higher. Overall, scab severity was more closely related to microbial function than to community composition within GS, and the functional similarity was higher in samples with L than in those with H.

**Fig. 4:**
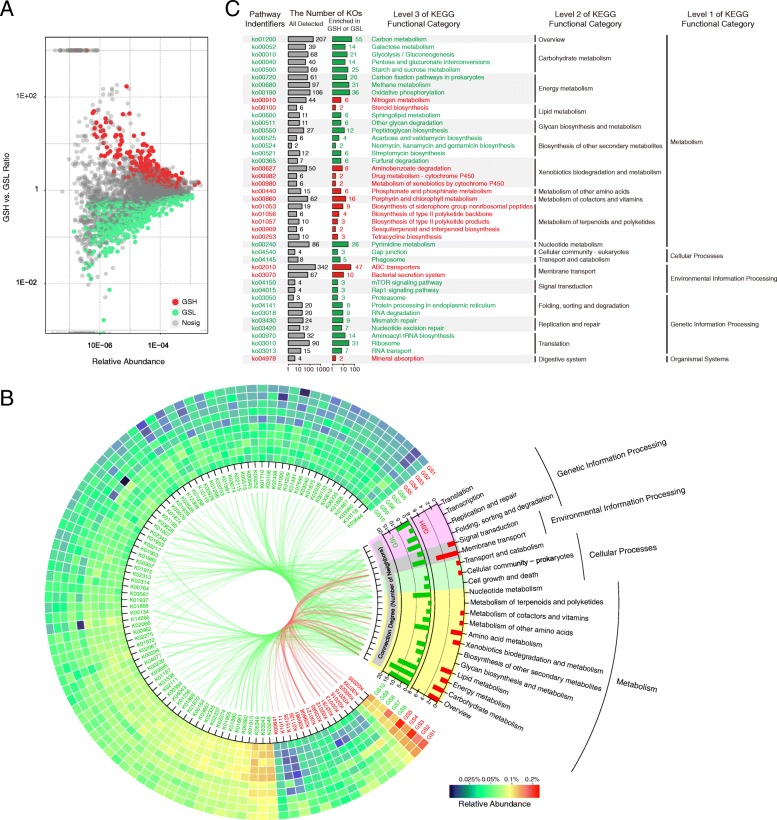
Significantly (*P* < 0.05) different **a**, **b** KO functional categories and **c** pathways between GSH and GSL. All KO functional categories are depicted in (**a**), and the differential KO functional categories were evaluated using the two-tailed Wilcoxon test. The abundant differential KO functional categories (relative abundance > 0.03%) are described in the heatmap (**b**), and the involved pathways are counted in the histogram and are linked by lines. KO functional categories that were significantly enriched in GSH or GSL were separately analyzed for KEGG pathway enrichment; those pathways that did not belong to microorganisms were removed; all significantly enriched pathways are described in (**c**). KO functional categories and pathways that were significantly enriched in GSH are shown in red; those significantly enriched in GSL are shown in green

We quantified and visualized the functional differences between GSH and GSL using a two-tailed Wilcoxon test, and a total of 240 and 561 significantly (two-tailed Wilcoxon test, *P* < 0.05) enriched KO functional categories were discovered in GSH and GSL, respectively (Fig. 4a). To clarify which KO functional categories were the most dominant among these differences, the differential KO functional categories with a relative abundance greater than 0.03% are described in Fig. 4b. Through this filter, 98 differential KO functional categories were retained, 16 of which were significantly enriched in GSH and 82 of which were significantly enriched in GSL. Notably, the relative abundance of K02035 (mean 00.19%) was the highest among all the differential KO functional categories, and K00799 was the second highest; they were both enriched in GSH. K02035, a peptide/nickel transport system substrate-binding protein, is a member of the “QS” pathway, and K00799 (*gst*, glutathione S-transferase; mean 0.14%) is supposed to participate in the “glutathione metabolism” and “xenobiotics biodegradation and metabolism” pathways. In the differential KO functional categories enriched in GSL, K03046 (*rpoC*, DNA-directed RNA polymerase subunit beta; mean 0.11%) was the most abundant KO functional category followed by K03043 (*rpoB*, DNA-directed RNA polymerase subunit beta; mean 0.11%). These functional categories are mainly involved in “purine metabolism,” “pyrimidine metabolism,” and “RNA polymerase” pathways. Moreover, we counted the pathways in which the abundant differential KO functional categories (relative abundance > 0.03%) were involved (Fig. 4b). In both GSH and GSL, “metabolism” pathways were the main components, but there were multiple pathways that belonged to “genetic information processing” in GSL but no such pathway in GSH.

To further explore the pathways in which all differential KO functional categories were involved, KO functional categories enriched in GSH or GSL were separately analyzed for KEGG pathway enrichment (Fig. 4c). The “nitrogen metabolism” pathway was significantly (*P* < 0.05) enriched in GSH. Concomitantly, effector genes and enzymes, including *nrtABC*, *nasDEF*, *cynAB*, *napB*, and formamidase, exhibited significantly increased relative abundance in the microbiome of GSH compared to that of GSL. These genes and enzymes are supposed to participate in the transport of nitrate and nitrite as well as the reduction of nitric oxide and the hydrolysis of formamide. The pathways involved in “drug metabolism—cytochrome P450” and the “metabolism of xenobiotics by cytochrome P450” were enriched in GSH and may play important roles in thaxtomin phytotoxin biosynthesis [38, 40]. The metabolism of some substances, such as aminobenzoate, drugs, cofactors, vitamins, terpenoids, and polyketides, was also enriched in GSH. Furthermore, the “steroid biosynthesis,” “ABC transporters,” and “bacterial secretion system” pathways were enriched in GSH. K03223, K03225, K03226, K03227, K03228, and K03229 are involved in the “bacterial secretion system” pathway and exhibited significantly increased relative abundances in GSH compared to GSL, and the genes that they matched, including *hrcQRSTU*, *sctLQRSTU*, and *yscLQRSTU*, encode type III secretion proteins. In contrast, the “carbohydrate metabolism” and “energy metabolism” pathways were significantly reduced in GSH compared to those in GSL. The biosynthesis pathways of some glycans and antibiotics, such as acarbose, streptomycin, validamycin, neomycin, kanamycin, and gentamicin, were also significantly depleted in GSH. Several pathways related to “genetic information processing,” such as “folding, sorting, and degradation”; “replication and repair”; and “translation”, were significantly depleted in GSH.

## Discussion

Soil microorganisms play important roles in plant disease occurrence and prevention [6–9], and microbial communities have been found to successfully suppress plant diseases in previous studies [10, 11]. Although some strains have been proven to reduce the incidence and/or severity of CS in previous studies [47–53], there has yet been no systematic study of the interactions between the soil microbiome and CS, so we systematically measured the relationships between soil microbiomes and CS using qPCR, bacterial 16S rRNA gene amplicon sequencing, and shotgun metagenomic sequencing.

Determining which soil-root system compartments are closely associated with CS is a matter of utmost importance, as it is related to the suitability of the surrounding soil for the continued planting of potatoes. Therefore, we investigated the relationships between CS and bacterial community composition in four soil-root system compartments, GS, RS, ZS, and FS, around potato plants. (i) High *txtAB* gene copy numbers were only detected in GS, while few or no *txtAB* gene copies were detected in the other compartments (Fig. 1a). (ii) The bacterial 16S rRNA gene copy numbers in GS were significantly different between H and L, while those in RS, ZS, and FS were not (Fig. 1b). (iii) Amplicon sequencing and alpha-diversity analysis indicated that marginal significant differences only existed between the H and L groups in GS, and no significant differences were detected in the other soil compartments (Fig. 1c). (iv) PCoA revealed that GSH and GSL could be distinguished according to scab severity, but there was no clear separation in RS, ZS, or FS (Fig. 1d–h). (v) The Wilcoxon test revealed 58 significantly differentiated genera in GS between H and L, far more than the 1 differentiated genus in RS, 5 differentiated genera in ZS, and 7 differentiated genera in FS (Table 1). In addition, a significant difference in the population levels of pathogenic *Streptomyces* between resistant and susceptible potato genotypes was found in the tubers but not in the roots or RS [66]. Similarly, no significant difference was found in the microbial community composition in soil within 10 cm of the central axis of the potato plant [67], which was equivalent to the ZS in the current study. Taken together, these results suggest that the studied GS bacterial community was closely associated with CS occurrence, while those of RS, ZS, and FS were not.

Microorganisms are one indicator of soil health [68–70]. In our study, marginally significantly higher bacterial alpha-diversity indices (OTU numbers, Chao1, and Shannon) were found in GSL than in GSH (Fig. 1c), and significantly lower *txtAB* gene abundance (Fig. 1a), lower bacterial abundance (Fig. 1b), higher co-occurrence network complexity of the bacterial community (Additional file 1: Figure S6), and higher microbial function similarity (Fig. 3) were also observed in GSL. Many previous studies have reported that higher soil bacterial community diversity is often associated with greater resistance to pathogen invasion [16, 17, 71], and the higher bacterial community diversity in GSL suggests that it may be more resistant to CS. Furthermore, community invasion resistance can be related to the complexity of microbial interaction networks [18, 19]. High microbial diversity can improve community stability [72, 73], which may explain why the more complex co-occurrence network of the bacterial community was present in samples with L (Additional file 1: Figure S6). Microbial function is more closely linked to environmental factors than community composition, and in a similar environment, microbial composition may vary greatly while microbial function remains similar [74–76]. Similar findings were found in our study (Fig. 3); the significant difference in microbial function similarity rather than composition similarity may indicate that scab severity was more closely related to microbial function than to community composition. Overall, the microbiome characteristics indicating soil health were high bacterial diversity and high co-occurrence network complexity. For CS, low thaxtomin biosynthetic gene *txtAB* abundance, low bacterial abundance, and high microbial function similarity in the GS microbiome could also be characteristics of soil health.

Plant pathogens have long been of concern because they can directly infect plants and cause diseases, but the role of the soil microbial community in disease development is of interest because it might directly or indirectly interact with plant pathogens and regulate disease occurrence [77–79]. The results of the correlation analysis revealed that some microorganisms are significantly associated with the scab severity level, EAA of pathogenic *Streptomyces*, and *txtAB* gene copy number (Fig. 2). *Variovorax* and *Stenotrophomonas* were significantly positively correlated with these three parameters and were the most abundant taxa in the bacterial community. *Variovorax* [80–82] and *Stenotrophomonas* [82, 83] have been reported to potentially hydrolyze cellulose, and cellobiose and cellotriose, the products of cellulose catabolism, have been shown to stimulate thaxtomin production [84, 85]. However, *S*. *scabies*, *S*. *acidiscabies*, and *S*. *turgidiscabies* did not show an ability to hydrolyze cellulose [84], so it was supposed that *Variovorax* and *Stenotrophomonas*, which have the potential for cellulose hydrolysis, may be responsible for inducing CS by stimulating thaxtomin production. In addition, the presence of cellulose 1,4-beta-cellobiosidase (EC:3.2.1.91; CBH2; CBHA; K19668) in our metagenomic KO profiling (Additional file 7) supported this hypothesis. The correlation analysis revealed that *Bacillus* was significantly negatively correlated with scab severity level and *txtAB* gene copy number (Fig. 2). Previous studies have reported that some microorganisms, such as *Bacillus*, *Pseudomonas*, non-pathogenic *Streptomyces*, and some fungi, can inhibit the growth or reduce the phytotoxicity of pathogenic *Streptomyces* [47–49, 52, 86]. *Bacillus* is one of the most famous biocontrol bacteria and it is widely used not only in the prevention and treatment of CS but also in the biological control of various plant diseases [87–89]. We also found that *Pseudomonas* was the most abundant genus with the highest relative abundance in GSL (Additional file 1: Table S3). Arseneault et al. demonstrated that a *Pseudomonas* strain, *Pseudomonas fluorescens* LBUM223, could directly alter the transcriptional activity of the *txtAB* gene in pathogenic *Streptomyces* under field conditions, contributing to disease control [49, 50]. Therefore, *Bacillus* and *Pseudomonas* may be two potential taxa functioning in the biocontrol of CS in our study. Overall, interaction networks may be formed between the microbial community and CS, and of the microorganisms that are significantly positively correlated with CS, some may act synergistically with pathogenic *Streptomyces* to cause the disease. Some microorganisms that are significantly negatively correlated with CS may also act as biocontrol agents in the consortium.

Both microbial community composition and microbial function within GS were closely associated with CS (Figs. 3 and 4). QS was recognized due to its ability to coordinate the expression of specific genes of multiple pathogens and to regulate pathogenic performance [90, 91], and K02035, a member of the “QS” pathway, exhibited the highest relative abundance of all the differential KO functional categories and was enriched in GSH. The higher relative abundance in GSH implies that some microorganisms may induce pathogenic *Streptomyces* to cause CS. The “steroid biosynthesis,” “ABC transporters,” and “bacterial secretion system” pathways were also enriched in GSH. Steroids can act as signal molecules that mediate communication between microorganisms and hosts [92, 93], and the “ABC transporters” and “bacterial secretion system” pathways also mediated communications between microorganisms and environments or other organisms [94–96]. Several genes involved in the “bacterial secretion system” pathway, such as *hrcQRSTU*, *sctLQRSTU*, and *yscLQRSTU*, encode type III secretion proteins; type III secretion systems (T3SSs) are essential for the pathogenicity (the ability to infect) of many pathogenic bacteria [97, 98]. The increased relative abundance of these genes in GSH may indicate a heightened ability to infest microbial communities. Furthermore, the “nitrogen metabolism,” “drug metabolism—cytochrome P450,” and “metabolism of xenobiotics by cytochrome P450” pathways were significantly enriched in GSH. Thaxtomins have been identified as having the basic structure l-4-nitrotryptophyl-l-phenylalanyl [99], and the biosynthetic pathway of ThxA involves nitric oxide synthase and cytochrome P450 [38–40]. Thus, the enrichment of the nitrogen metabolism and cytochrome P450 pathways in GSH might contribute to ThxA biosynthesis. Moreover, the biosynthesis pathways of some antibiotics, such as streptomycin, validamycin, neomycin, kanamycin, and gentamicin, were significantly enriched in GSL; these antibiotics may play some role in controlling the biomass and composition of the microbial community, which may be one of the reasons why the bacterial abundance in GSH was higher than that in GSL. In short, the occurrence of CS was accompanied by an increase in the relative abundance of multiple pathogenicity-related genes in the soil microbiome.

Both metagenomic sequencing and bacterial 16S amplicon sequencing were performed to explore the GS microbiome in the present study. Amplicon sequencing can reveal microbial community composition, while metagenomic sequencing can reveal microbial community composition as well as community function. For bacterial community composition at the genus level, similar amplicon and metagenomic sequencing clustering patterns were described by PCoA (Additional file 1: Figure S7), demonstrating that the community similarity relationships identified by the two techniques were relatively consistent. While amplicon sequencing is unable to detect microbial community function, our metagenomic data revealed some functions associated with scab severity (Fig. 4). Furthermore, due to sequencing technology limitations, amplicon sequencing failed to identify *Streptomyces* to the species level, but analyzing the taxonomic profile of our metagenomic data revealed some species of *Streptomyces*, two of which were possible scab pathogens. Since metagenomic sequencing is relatively expensive, we first performed amplicon sequencing for all samples and then selected prominent samples for metagenomic sequencing; this strategy has been adopted in many previous studies [100–102]. In addition, we estimated the absolute and relative abundance of microbial species in the GS metagenomic profiles. Previous studies have mostly been based on the relative abundance of microbial profiles, but there are now ways to estimate absolute abundance from relative abundance [64, 103, 104]. We assessed the correlations between the entire *Streptomyces* genus and the severity of CS, and the results indicated that the EAA of the entire *Streptomyces* genus was significantly correlated with CS severity (Fig. 2; Spearman, ρ = 0.89, *P* = 0.0005), while the relative abundance was not (Spearman, ρ = 0.54, *P* = 0.11). Consistently, the scab severity level was more related to the EAA of pathogenic *Streptomyces* (Spearman, ρ = 0.89, *P* = 0.0005) than to the relative abundance (Spearman, ρ = 0.66, *P* = 0.036). These results demonstrate that absolute abundance better reflects the CS severity than relative abundance.

As the important members of the soil-root system, plants, microorganisms, and the environment can interact with each other. We noticed differences in the soil physicochemical characteristics (Additional file 1: Table S1) and spatial separation (Additional file 1: Figure S1) of the sample sites between the H and L groups, so it was necessary to verify whether the relationships between the soil microbiome and scab occurrence remain valid with interference from these spatial factors. Despite spatial separation and soil physicochemical differences, there were no significant differences in bacterial 16S copy numbers (Fig. 1b), diversity (Fig. 1c, Additional file 1: Table S2), and compositions (Fig. 1g, h) between the H and L groups in ZS and FS. Thus, the differences in the microbial community characteristics (16S copy number, diversity, community composition, and function) of GS between H and L are more likely to be directly related to CS severity. The spatial effects and soil physicochemical characteristics are not the primary factors responsible for directly shaping the microbial community in this study.

Based on microbial composition and function profiles, we proposed a hypothetical model of the effect of the soil microbiome on CS (Additional file 1: Figure S8). In addition to the direct effects of pathogenic *Streptomyces* on the occurrence of CS, some soil microorganisms may also directly or indirectly promote or suppress the occurrence of CS. Through the “ABC transporters,” “bacterial secretion system,” and “QS” pathways, some microorganisms can form an interacting consortium with pathogenic *Streptomyces* to promote the expression of thaxtomin biosynthetic genes and help pathogenic *Streptomyces* to infect their host. Cellulose-hydrolyzing strains, such as *Variovorax* and *Stenotrophomonas*, may induce CS by generating cellobiose and cellotriose to stimulate pathogenic *Streptomyces* to synthesize ThxA, and “nitrogen metabolism” and cytochrome P450 from some uncertain microorganisms may contribute to ThxA biosynthesis. In contrast, the biosynthesis of antibiotics can control the biomass of the microbial community and/or reduce the population of pathogenic *Streptomyces*, thus indirectly and/or directly delaying the occurrence of CS. There were also some taxa, such as *Bacillus* and *Pseudomonas*, that could inhibit the biosynthesis of ThxA and/or reduce the population of pathogenic *Streptomyces*, and non-pathogenic *Streptomyces* could also reduce the population of pathogenic *Streptomyces* due to niche overlap. This model provides some basic insights into and ideas for clarifying the occurrence of potato CS. However, the hypothetical model remains incomplete, and additional microorganisms and functions associated with CS need to be discovered. Furthermore, more studies verifying differentiated microorganisms and functions need to be implemented in the future.

## Conclusions

This study aimed to address the interactions between the soil microbiome and CS, and it confirmed that the microbial community composition and function of GS are associated with CS severity. Low *txtAB* gene abundance, low bacterial abundance, high diversity, high co-occurrence network complexity, and high community function similarity in GS were indicators of low potato CS severity. The GS microbiome associated with potatoes with high scab severity contained pathogenicity-related gene profiles, and several genes involved in the “ABC transporters,” “bacterial secretion system,” “QS,” “nitrogen metabolism” pathways, and some metabolism by cytochrome P450 were significantly enriched in the H group. In contrast, the biosynthetic pathways of some antibiotics were significantly enriched in the L group. Our study broadens the understanding of the relationships between the occurrence of CS and the soil microbiome and provides novel insights into CS occurrence.

## Additional files

Additional file 1: **Figure S1.** Summary of sampling in this study. The sampling field (34.248727°N, 119.816724°E, 22.9 m a.s.l.) was located in Jiaozhou City in Shandong Province, China. **Figure S2.** Significantly (*P* < 0.05) differentiated bacterial taxa between GSH and GSL as evaluated by the linear discriminant analysis effect size (LEfSe) with LDA scores > 2. **Figure S3.** Significantly (*P* < 0.05) differentiated bacterial genera between GSH and GSL as evaluated using the linear discriminant analysis effect size (LEfSe) with LDA scores > 2 and a two-tailed Wilcoxon test. **Figure S4.** Phylogenetic tree for 16S rRNA gene sequences of the isolated strains using the neighbor-joining method. **Figure S5.** The conversion of the EAA of pathogenic *Streptomyces* and the copy numbers of the thaxtomin biosynthetic gene *txtAB*. **Figure S6.** The co-occurrence network interactions of metagenomic bacterial communities. **Figure S7.** (A) 16S amplicon sequencing and (B) metagenomic sequencing exhibited similar bacterial community dissimilarity clustering patterns in GS. **Figure S8.** Hypothetical model of the effect of the soil microbiome on CS. **Table S1.** Soil physicochemical characteristics of ZS and FS. **Table S2.** Bacterial alpha-diversity indices of GS, RS, ZS and FS based on the rarefied OTUs at a depth of 29,718 sequences per sample. **Table S3.** The genera with the highest relative abundance (top 10) in the bacterial community (amplicon sequencing). **Table S4.** 18 *Streptomyces* species aligned against the NR database that were possible scab pathogens. **Table S5.** Strains isolated from the culture experiment with the phylogenetic similarity to pathogenic *Streptomyces. (DOC 2829 kb)*

Additional file 2: Command lines of bacterial 16S amplicon sequencing analysis. (TXT 2 kb)

Additional file 3: Sequence read numbers of the bacterial OTUs profiling (sequenced on a MiSeq PE250 sequencer at a depth of 29,718 sequences per sample). (XLS 882 kb)

Additional file 4: Relative abundance of the metagenomic microbial taxonomic profiling (at genus level) (XLS 356 kb)

Additional file 5: Relative abundance of the metagenomic bacterial taxonomic profiling (at genus level) (separated from the microbial taxonomic profiling) (XLS 355 kb)

Additional file 6: 16S rRNA gene sequences of the 12 isolated *Streptomyces* stains. (TXT 15 kb)

Additional file 7: Estimated absolute abundance (EAA) of the metagenomic bacterial taxonomic profiling (at genus level) (XLS 354 kb)

Additional file 8: Relative abundance of the metagenomic microbial function profiling (KEGG orthology function category) (XLS 928 kb)

## Abbreviations

CS: Common scab
EAA: Estimated absolute abundance
FS: Furrow soil
GS: Geocaulosphere soil
H: (the group with) High common scab severity
KO: KEGG orthology
L: (the group with) Low common scab severity
RS: Rhizosphere soil
ThxA: Thaxtomin A
ZS: Root-zone soil
