# Supplementary material for: The occurrence of potato common scab correlates with the community composition and function of the geocaulosphere soil microbiome
Source: Microbiome. 2019 Feb 1;7:14. doi: 10.1186/s40168-019-0629-2 (PMC6359780; doi:10.1186/s40168-019-0629-2)
Supplement: Supplementary file 1 — Figure S1. Summary of sampling in this study. The sampling field (34.248727°N, 119.816724°E, 22.9 m a.s.l.) was located in Jiaozhou City in Shandong Province, China. Figure S2. Significantly (P < 0.05) differentiated bacterial taxa between GSH and GSL as evaluated by the linear discriminant analysis effect size (LEfSe) with LDA scores > 2. Figure S3. Significantly (P < 0.05) differentiated bacterial genera between GSH and GSL as evaluated using the linear discriminant analysis effect size (LEfSe) with LDA scores > 2 and a two-tailed Wilcoxon test. Figure S4. Phylogenetic tree for 16S rRNA gene sequences of the isolated strains using the neighbor-joining method. Figure S5. The conversion of the EAA of pathogenic Streptomyces and the copy numbers of the thaxtomin biosynthetic gene txtAB. Figure S6. The co-occurrence network interactions of metagenomic bacterial communities. Figure S7. (A) 16S amplicon sequencing and (B) metagenomic sequencing exhibited similar bacterial community dissimilarity clustering patterns in GS. Figure S8. Hypothetical model of the effect of the soil microbiome on CS. Table S1. Soil physicochemical characteristics of ZS and FS. Table S2. Bacterial alpha-diversity indices of GS, RS, ZS and FS based on the rarefied OTUs at a depth of 29,718 sequences per sample. Table S3. The genera with the highest relative abundance (top 10) in the bacterial community (amplicon sequencing). Table S4. 18 Streptomyces species aligned against the NR database that were possible scab pathogens. Table S5. Strains isolated from the culture experiment with the phylogenetic similarity to pathogenic Streptomyces. (DOC 2829 kb) [file 40168_2019_629_MOESM1_ESM.doc]

**Supplementary Information**

1. **Supplemental Figures**

**Figure S1** Summary of sampling in this study. The sampling field (34.248727°N, 119.816724°E, 22.9 m a.s.l.) was located in Jiaozhou City in Shandong Province, China. Ten potato plants were selected and divided into two groups (H: No. 1 to 5, the group with high scab severity levels; L: No. 6 to 10, the group with low scab severity levels) according to scab severity. Four soil-root system compartments (geocaulosphere soil (GS), rhizosphere soil (RS), root-zone soil (ZS) and furrow soil (FS)) were collected and analysed for each plant.

**Figure S2** Significantly (*P* < 0.05) differentiated bacterial taxa between GSH and GSL as evaluated by the linear discriminant analysis effect size (LEfSe) with LDA scores > 2. For each taxon (circle), the colour denotes the significantly (*P* < 0.05) higher relative abundance of the taxon in the corresponding group (red: GSH; green: GSL; yellow: not significantly enriched in any group).

**Figure S3** Significantly (*P* < 0.05) differentiated bacterial genera between GSH and GSL as evaluated using the linear discriminant analysis effect size (LEfSe) with LDA scores > 2 and a two-tailed Wilcoxon test. The colour denotes the significantly (*P* < 0.05) higher relative abundance of the genera in the corresponding group (red: GSH; green: GSL; grey: not significantly enriched in any group). All differentiated (*P* < 0.05) bacterial genera evaluated by both the LEfSe and Wilcoxon test are shown in (B).

**Figure S4** Phylogenetic tree for 16S rRNA gene sequences of the isolated strains using the neighbour-joining method. The percentage of replicate trees in which the associated taxa clustered together in the bootstrap test (1000 replicates) are shown next to the branches. The tree is drawn to scale, with branch lengths in the same units as those of the evolutionary distances used to infer the phylogenetic tree. The evolutionary distances were computed using the Jukes-Cantor method and are in the units of the number of base substitutions per site. The analysis involved 12 isolated sequences and 11 reference sequences from an NCBI 16S ribosomal RNA sequence (Bacteria and Archaea) database.

**Figure S5** The conversion of the EAA of pathogenic *Streptomyces* and the copy numbers of the thaxtomin biosynthetic gene *txtAB*. The error bars indicate the SD of three replicates. GSH is shown in red, and GSL is shown in green.

**Figure S6** The co-occurrence network interactions of metagenomic bacterial communities. (A) GSH; (B) GSL. A connection represents a significant correlation (Spearman, |ρ| > 0.8, *P* < 0.05). Red lines represent positive connections, while blue lines represent negative connections. The size of each node is proportional to the EAA of each genus. The same node colours represent nodes belonging to the same phylum.

**Figure S7** (A) 16S amplicon sequencing and (B) metagenomic sequencing exhibited similar bacterial community dissimilarity clustering patterns in GS. The dissimilarity of bacterial profiles at the genus level was visualized by PCoA based on the Bray-Curtis metric. GSH is shown in red and is denoted by triangles; GSL is shown in green and is denoted by circles.

**Figure S8** Hypothetical model of the effect of the soil microbiome on CS. Rounded rectangles denote microorganism strains, and circles denote substances. Red lines indicate promotion; green lines indicate inhibition; and grey lines indicate the production of substances.

1. **Supplemental Tables**

**Table S1** Soil physicochemical characteristics of ZS and FS

**Table S2** Bacterial alpha-diversity indices of GS, RS, ZS and FS based on the rarefied OTUs at a depth of 29,718 sequences per sample

**Table S3** The genera with the highest relative abundance (top 10) in the bacterial community (amplicon sequencing)

**Table S4** 18 *Streptomyces* species aligned against the NR database that were possible scab pathogens

**Table S5** Strains isolated from the culture experiment with the phylogenetic similarity to pathogenic *Streptomyces*

1. **Supplemental Figures**


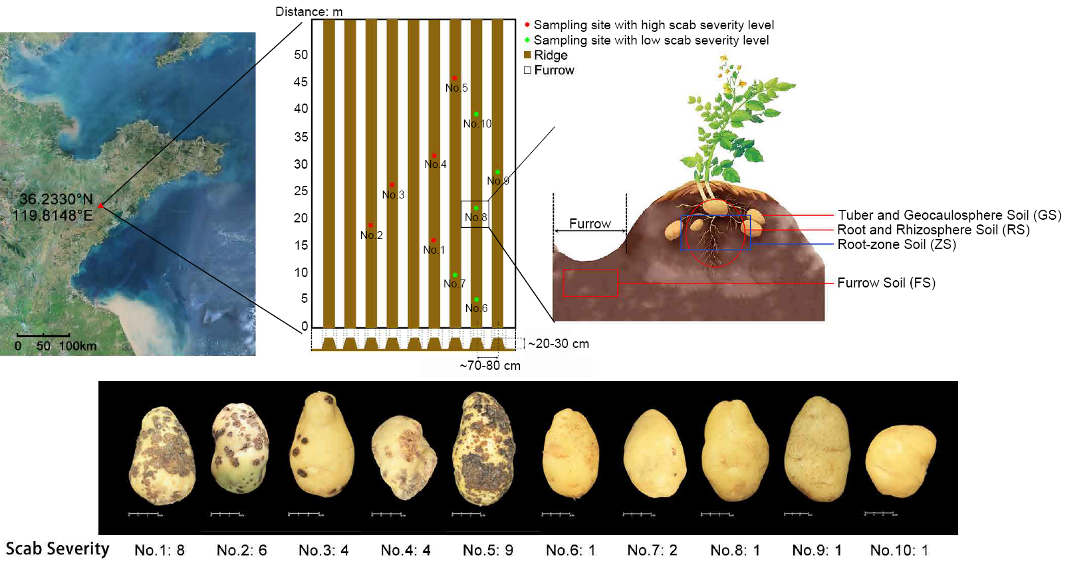


**Fig. S1** Summary of sampling in this study. The sampling field (34.248727°N, 119.816724°E, 22.9 m a.s.l.) was located in Jiaozhou City in Shandong Province, China. Ten potato plants were selected and divided into two groups (H: No. 1 to 5, the group with high scab severity levels; L: No. 6 to 10, the group with low scab severity levels) according to scab severity. Four soil-root system compartments (geocaulosphere soil (GS), rhizosphere soil (RS), root-zone soil (ZS) and furrow soil (FS)) were collected and analysed for each plant.


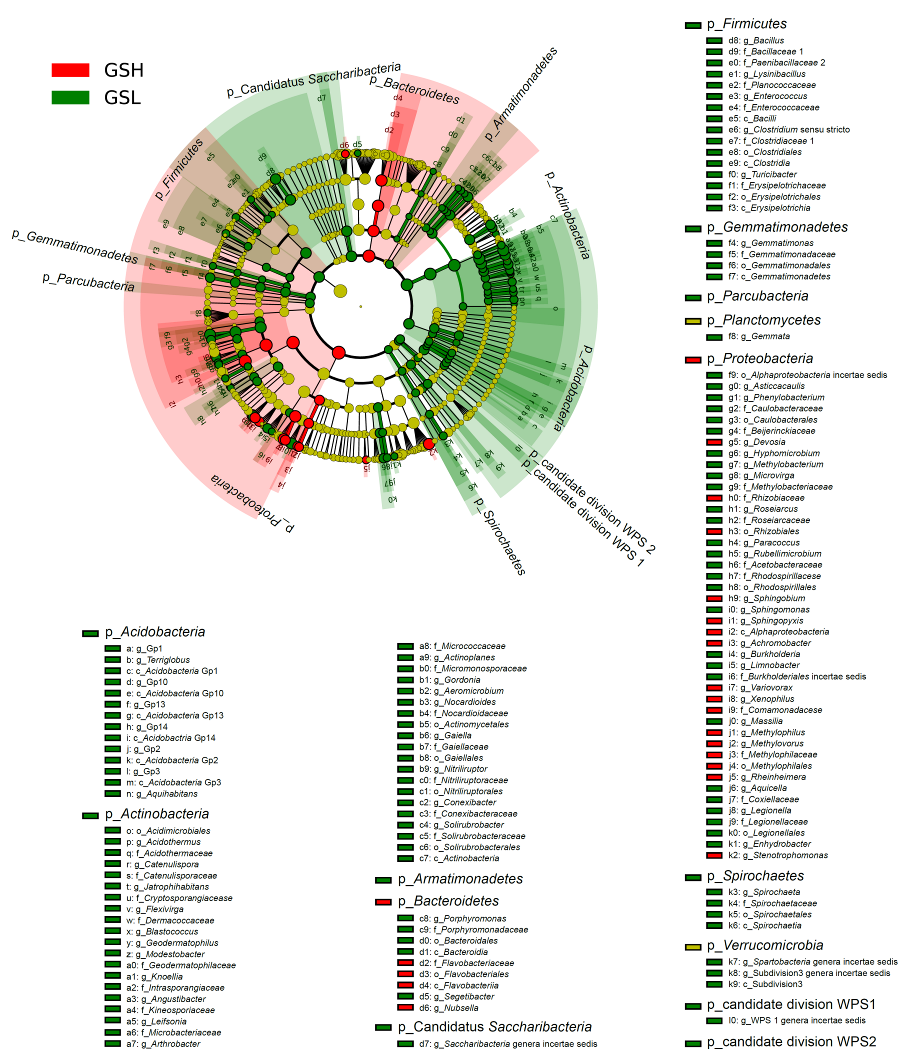


**Fig. S2** Significantly (*P* < 0.05) differentiated bacterial taxa between GSH and GSL as evaluated by the linear discriminant analysis effect size (LEfSe) with LDA scores > 2. For each taxon (circle), the colour denotes the significantly (*P* < 0.05) higher relative abundance of the taxon in the corresponding group (red: GSH; green: GSL; yellow: not significantly enriched in any group).


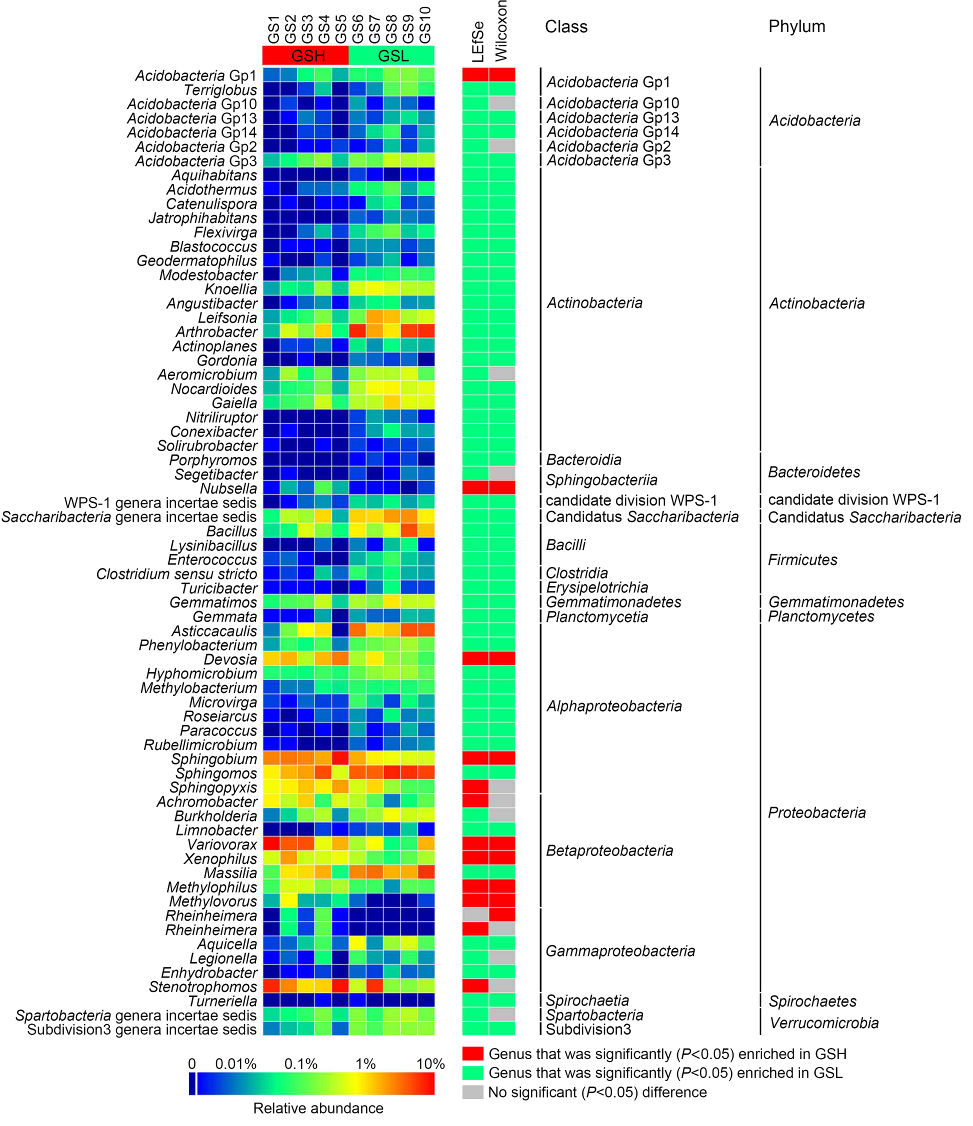


**Fig. S3** Significantly (*P* < 0.05) differentiated bacterial genera between GSH and GSL as evaluated using the linear discriminant analysis effect size (LEfSe) with LDA scores > 2 and a two-tailed Wilcoxon test. The colour denotes the significantly (*P* < 0.05) higher relative abundance of the genera in the corresponding group (red: GSH; green: GSL; grey: not significantly enriched in any group). All differentiated (*P* < 0.05) bacterial genera evaluated by both the LEfSe and Wilcoxon test are shown in (B).

**Fig. S4** Phylogenetic tree for 16S rRNA gene sequences of the isolated strains using the neighbour-joining method. The percentage of replicate trees in which the associated taxa clustered together in the bootstrap test (1000 replicates) are shown next to the branches. The tree is drawn to scale, with branch lengths in the same units as those of the evolutionary distances used to infer the phylogenetic tree. The evolutionary distances were computed using the Jukes-Cantor method and are in the units of the number of base substitutions per site. The analysis involved 12 isolated sequences and 11 reference sequences from an NCBI 16S ribosomal RNA sequence (Bacteria and Archaea) database.


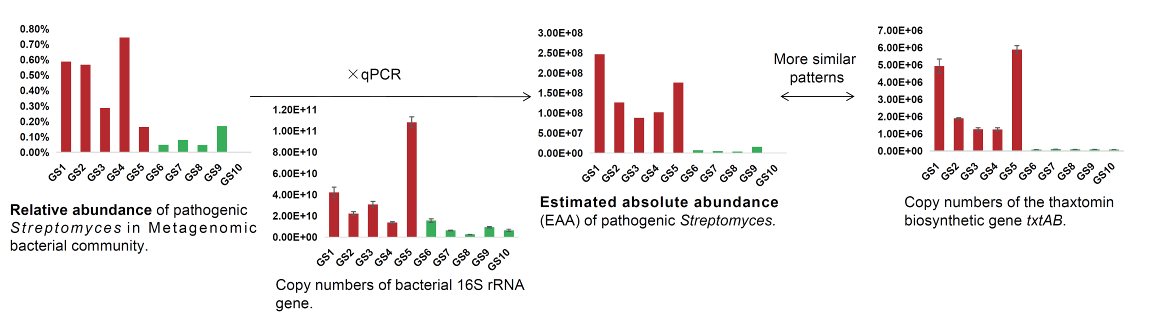


**Fig. S5** The conversion of the EAA of pathogenic *Streptomyces* and the copy numbers of the thaxtomin biosynthetic gene *txtAB*. The error bars indicate the SD of three replicates. GSH is shown in red, and GSL is shown in green.


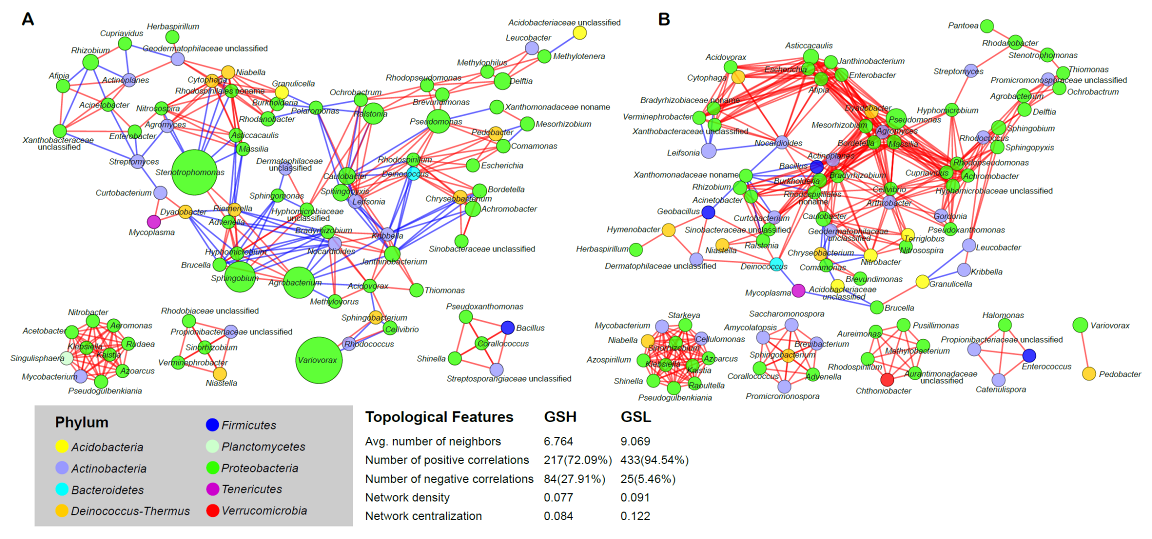


**Fig. S6** The co-occurrence network interactions of metagenomic bacterial communities. (A) GSH; (B) GSL. A connection represents a significant correlation (Spearman, |ρ| > 0.8, *P* < 0.05). Red lines represent positive connections, while blue lines represent negative connections. The size of each node is proportional to the EAA of each genus. The same node colours represent nodes belonging to the same phylum.


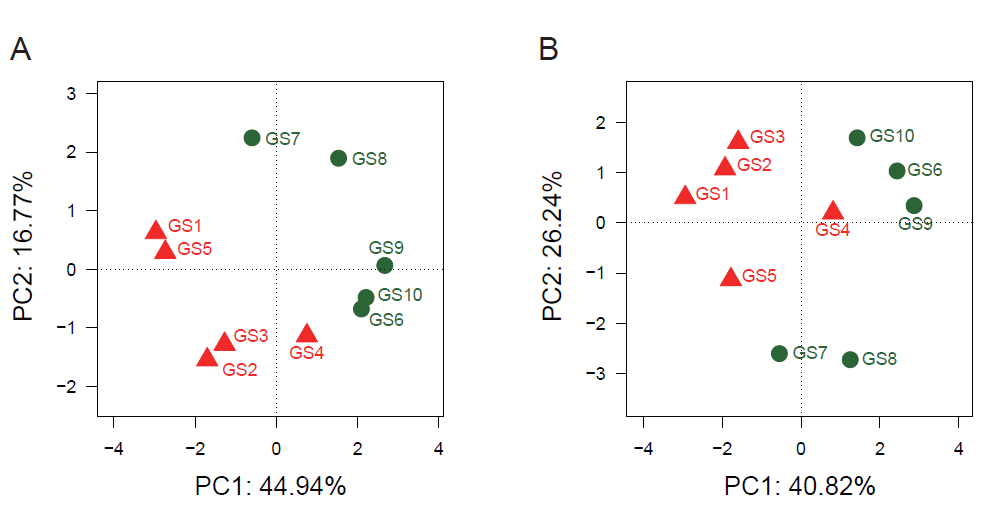


**Fig. S7** (A) 16S amplicon sequencing and (B) metagenomic sequencing exhibited similar bacterial community dissimilarity clustering patterns in GS. The dissimilarity of bacterial profiles at the genus level was visualized by PCoA based on the Bray-Curtis metric. GSH is shown in red and is denoted by triangles; GSL is shown in green and is denoted by circles.

**Fig. S8** Hypothetical model of the effect of the soil microbiome on CS. Rounded rectangles denote microorganism strains, and circles denote substances. Red lines indicate promotion; green lines indicate inhibition; and grey lines indicate the production of substances.

1. **Supplemental Tables**

**Tab. S1** Soil physicochemical characteristics of ZS and FS

| Sample ID | Group | pH | TC (g/Kg) | OM (g/kg) | TN (g/kg) | NO_3_^-^-N (mg/kg) | NH_4_^+^-N (mg/kg) | AK (mg/kg) | AP (mg/kg) | AS (mg/kg) | EC (μS/cm) |
| --- | --- | --- | --- | --- | --- | --- | --- | --- | --- | --- | --- |
| ZS1 | ZSH | 5.10 ± 0.03 | 8.39 ± 0.12 | 11.80 ± 0.81 | 1.53 ± 0.05 | 46.27 ± 9.78 | 13.57 ± 3.30 | 170.95 ± 4.91 | 149.72 ± 1.66 | 145.76 ± 7.09 | 328.00 ± 18.73 |
| ZS2 |  | 5.65 ± 0.04 | 9.09 ± 0.13 | 13.27 ± 2.21 | 1.47 ± 0.01 | 31.12 ± 4.99 | 12.25 ± 1.56 | 170.71 ± 4.88 | 116.47 ± 8.49 | 140.98 ± 5.26 | 279.33 ± 8.14 |
| ZS3 |  | 5.21 ± 0.02 | 8.63 ± 0.05 | 12.87 ± 0.78 | 1.58 ± 0.05 | 40.04 ± 6.64 | 11.50 ± 2.13 | 158.18 ± 2.48 | 128.51 ± 7.39 | 92.68 ± 2.73 | 294.00 ± 15.39 |
| ZS4 |  | 4.89 ± 0.03 | 7.93 ± 0.23 | 13.87 ± 0.12 | 1.64 ± 0.06 | 58.98 ± 8.22 | 11.13 ± 2.94 | 216.02 ± 14.35 | 156.67 ± 6.94 | 149.04 ± 9.48 | 426.67 ± 9.87 |
| ZS5 |  | 4.32 ± 0.03 | 7.61 ± 0.10 | 13.23 ± 0.25 | 1.66 ± 0.09 | 70.97 ± 7.52 | 22.62 ± 4.42 | 254.74 ± 10.70 | 141.95 ± 4.17 | 393.04 ± 31.27 | 858.33 ± 14.29 |
| ZS6 | ZSL | 5.66 ± 0.04 | 7.57 ± 0.01 | 11.23 ± 0.60 | 1.84 ± 0.04 | 40.01 ± 5.33 | 9.12 ± 2.69 | 145.41 ± 1.42 | 135.55 ± 3.23 | 100.74 ± 7.90 | 264.67 ± 13.20 |
| ZS7 |  | 5.11 ± 0.08 | 7.62 ± 0.17 | 10.43 ± 0.08 | 1.75 ± 0.06 | 50.92 ± 7.15 | 8.97 ± 4.65 | 146.39 ± 0.27 | 147.22 ± 4.97 | 130.94 ± 3.06 | 392.67 ± 12.10 |
| ZS8 |  | 4.82 ± 0.05 | 8.43 ± 0.07 | 12.13 ± 0.22 | 1.68 ± 0.07 | 53.58 ± 9.77 | 12.11 ± 5.62 | 231.64 ± 12.65 | 113.37 ± 4.49 | 233.40 ± 10.44 | 488.33 ± 7.09 |
| ZS9 |  | 5.28 ± 0.04 | 7.91 ± 0.13 | 11.92 ± 0.63 | 1.64 ± 0.05 | 40.51 ± 5.69 | 10.73 ± 4.53 | 212.36 ± 8.37 | 141.03 ± 7.90 | 131.23 ± 1.62 | 329.67 ± 11.15 |
| ZS10 |  | 4.96 ± 0.08 | 7.74 ± 0.11 | 13.03 ± 0.47 | 1.45 ± 0.05 | 45.06 ± 6.56 | 10.80 ± 2.90 | 209.92 ± 12.48 | 153.87 ± 6.49 | 152.29 ± 7.48 | 376.33 ± 25.03 |
| Wilcoxon | ZSH vs. ZSL | *P* = 0.69 | *P* = 0.22 | *P* = 0.10 | *P* = 0.25 | *P* = 0.84 | ***P* = 0.03** | *P* = 0.70 | *P* = 0.84 | *P* = 0.84 | *P* = 1 |
| FS2 | FSH | 6.33 ± 0.02 | 8.97 ± 0.06 | 13.69 ± 1.34 | 1.62 ± 0.05 | 64.44 ± 23.45 | 15.13 ± 8.61 | 174.21 ± 2.25 | 119.75 ± 2.80 | 94.30 ± 4.65 | 309.00 ± 3.61 |
| FS3 |  | 4.99 ± 0.02 | 8.75 ± 0.04 | 13.81 ± 1.95 | 1.66 ± 0.05 | 78.10 ± 3.77 | 14.09 ± 2.96 | 175.10 ± 5.50 | 133.65 ± 3.48 | 284.30 ± 6.57 | 975.67 ± 43.09 |
| FS4 |  | 5.00 ± 0.05 | 8.12 ± 0.11 | 13.67 ± 1.63 | 1.61 ± 0.08 | 60.75 ± 10.92 | 9.41 ± 1.38 | 220.82 ± 10.54 | 148.85 ± 7.40 | 132.57 ± 14.03 | 502.33 ± 29.74 |
| FS5 |  | 4.79 ± 0.02 | 7.40 ± 0.06 | 12.63 ± 1.90 | 1.56 ± 0.05 | 61.53 ± 10.99 | 8.18 ± 0.70 | 145.90 ± 0.57 | 119.32 ± 8.08 | 170.31 ± 17.95 | 472.00 ± 12.49 |
| FS6 | FSL | 4.94 ± 0.01 | 7.52 ± 0.02 | 12.50 ± 0.07 | 1.57 ± 0.06 | 64.55 ± 14.01 | 10.86 ± 1.38 | 135.89 ± 7.46 | 162.80 ± 3.27 | 151.16 ± 9.34 | 529.00 ± 2.65 |
| FS7 |  | 4.91 ± 0.06 | 7.49 ± 0.12 | 11.70 ± 0.57 | 1.58 ± 0.02 | 70.55 ± 10.65 | 8.96 ± 1.52 | 203.49 ± 2.23 | 155.87 ± 2.68 | 125.77 ± 4.53 | 444.33 ± 23.46 |
| FS8 |  | 4.76 ± 0.02 | 7.47 ± 0.12 | 12.94 ± 0.52 | 1.41 ± 0.02 | 55.10 ± 22.08 | 7.86 ± 3.29 | 183.40 ± 3.23 | 138.20 ± 3.30 | 145.97 ± 6.29 | 473.67 ± 11.50 |
| FS9 |  | 6.81 ± 0.03 | 7.50 ± 0.10 | 12.89 ± 1.04 | 1.89 ± 0.39 | 43.86 ± 41.55 | 6.82 ± 1.02 | 139.31 ± 2.54 | 112.82 ± 2.01 | 86.01 ± 9.71 | 160.03 ± 3.02 |
| FS10 |  | 4.59 ± 0.03 | 7.57 ± 0.02 | 13.00 ± 0.85 | 1.98 ± 0.42 | 85.65 ± 5.88 | 9.11 ± 1.04 | 300.29 ± 16.46 | 166.45 ± 3.62 | 327.71 ± 19.67 | 953.33 ± 32.25 |
| Wilcoxon | FSH vs. FSL | *P* = 0.41 | *P* = 0.29 | *P* = 0.11 | *P* = 1 | *P* = 1 | *P* = 0.19 | *P* = 1 | *P* = 0.29 | *P* = 0.90 | *P* = 0.90 |
| Wilcoxon | H vs. L  (ZS and FS combined) | *P* = 0.90 | *P* = 0.02 | *P* = 0.01 | *P* = 0.31 | *P* = 0.66 | *P* = 0.01 | *P* = 0.84 | *P* = 0.45 | *P* = 0.72 | *P* = 0.97 |

Each characteristic was detected with 3 technical replicates, and the mean ± SD is displayed in the table. Differences in the characteristics were identified using the two-tailed Wilcoxon test between the H and L groups. TC: soil total carbon; OM: organic matter; TN: total nitrogen; AK: available potassium; AP: available phosphorus; AS: available sulphur; EC: electrical conductivity.

**Tab. S2** Bacterial alpha-diversity indices of GS, RS, ZS and FS based on the rarefied OTUs at a depth of 29,718 sequences per sample

| **Sample ID** | **OTU** | **Ace** | **Chao1** | **Shannon** | **Simpson** | **Coverage** | **Sample ID** | **OTU** | **Ace** | **Chao1** | **Shannon** | **Simpson** | **Coverage** |
| --- | --- | --- | --- | --- | --- | --- | --- | --- | --- | --- | --- | --- | --- |
| GS1 | 380 | 973 | 675 | 2.82 | 0.1443 | 0.99 |  |  |  |  |  |  |  |
| GS2 | 659 | 1468 | 1070 | 3.79 | 0.0558 | 0.99 | FS2 | 2009 | 2331 | 2361 | 6.17 | 0.0121 | 0.99 |
| GS3 | 754 | 1445 | 1122 | 3.66 | 0.0675 | 0.99 | FS3 | 1568 | 1971 | 1997 | 5.57 | 0.0169 | 0.99 |
| GS4 | 1118 | 1594 | 1522 | 5.01 | 0.0187 | 0.99 | FS4 | 1713 | 2054 | 2064 | 5.67 | 0.0213 | 0.99 |
| GS5 | 434 | 1580 | 872 | 3.24 | 0.0779 | 0.99 | FS5 | 1553 | 1933 | 1964 | 5.72 | 0.0092 | 0.99 |
| GS6 | 1337 | 1864 | 1825 | 4.84 | 0.0329 | 0.98 | FS6 | 1937 | 2238 | 2229 | 6.00 | 0.0130 | 0.99 |
| GS7 | 778 | 1370 | 1218 | 4.37 | 0.0290 | 0.99 | FS7 | 1683 | 1995 | 2026 | 5.85 | 0.0121 | 0.99 |
| GS8 | 1096 | 1529 | 1491 | 4.67 | 0.0310 | 0.99 | FS8 | 1719 | 2108 | 2148 | 5.93 | 0.0095 | 0.99 |
| GS9 | 1205 | 1958 | 1715 | 4.92 | 0.0212 | 0.99 | FS9 | 1972 | 2235 | 2258 | 6.21 | 0.0131 | 0.99 |
| GS10 | 1087 | 1561 | 1580 | 4.60 | 0.0280 | 0.99 | FS10 | 1742 | 2089 | 2129 | 5.77 | 0.0147 | 0.99 |
| RS1 | 403 | 869 | 691 | 2.84 | 0.1735 | 0.99 | ZS1 | 1654 | 1983 | 1941 | 5.85 | 0.0101 | 0.99 |
| RS2 | 1005 | 1423 | 1389 | 4.71 | 0.0260 | 0.99 | ZS2 | 1912 | 2209 | 2252 | 6.13 | 0.0094 | 0.99 |
| RS3 | 382 | 924 | 757 | 1.85 | 0.4271 | 0.99 | ZS3 | 1495 | 1827 | 1823 | 5.60 | 0.0141 | 0.99 |
| RS4 | 961 | 1664 | 1441 | 4.79 | 0.0220 | 0.99 | ZS4 | 1712 | 2077 | 2074 | 5.54 | 0.0181 | 0.99 |
| RS5 | 247 | 449 | 375 | 2.49 | 0.1846 | 1.00 | ZS5 | 1208 | 1509 | 1531 | 4.97 | 0.0430 | 0.99 |
| RS6 | 456 | 879 | 802 | 3.22 | 0.1197 | 0.99 | ZS6 | 1912 | 2229 | 2229 | 6.02 | 0.0109 | 0.99 |
| RS7 | 433 | 885 | 671 | 3.20 | 0.1081 | 0.99 | ZS7 | 1647 | 1972 | 1985 | 5.80 | 0.0117 | 0.99 |
| RS8 | 396 | 831 | 633 | 2.54 | 0.2319 | 0.99 | ZS8 | 1519 | 1918 | 1919 | 5.51 | 0.0180 | 0.99 |
| RS9 | 709 | 1308 | 1087 | 3.20 | 0.1719 | 0.99 | ZS9 | 1808 | 2152 | 2147 | 5.83 | 0.0135 | 0.99 |
| RS10 | 964 | 1740 | 1408 | 4.21 | 0.0550 | 0.99 | ZS10 | 1628 | 2046 | 2036 | 5.37 | 0.0234 | 0.98 |

**Tab. S3** The genera with the highest relative abundance (top 10) in the bacterial community (amplicon sequencing)

| **Rank** |  | **Genus** | **Relative Abundance** |  | **Genus** | **Relative Abundance** |  | **Genus** | **Relative Abundance** |  | **Genus** | **Relative Abundance** |
| --- | --- | --- | --- | --- | --- | --- | --- | --- | --- | --- | --- | --- |
| **1** | GSH | *Rhizobium* | 7.64% | GSL | *Pseudomonas* | 7.07% | RSH | *Pseudomonas* | 14.77% | RSL | *Pseudomonas* | 22.37% |
| **2** |  | *Variovorax* | 5.60% |  | *Sphingomonas* | 7.02% |  | *Flavobacterium* | 4.27% |  | *Sphingobium* | 6.87% |
| **3** |  | *Chryseobacterium* | 5.59% |  | *Arthrobacter* | 5.77% |  | *Sphingobacterium* | 3.42% |  | *Bacillus* | 4.95% |
| **4** |  | *Stenotrophomonas* | 5.46% |  | *Massilia* | 3.99% |  | *Sphingobium* | 3.38% |  | *Arthrobacter* | 3.29% |
| **5** |  | *Sphingobium* | 4.95% |  | *Asticcacaulis* | 3.69% |  | *Stenotrophomonas* | 3.00% |  | *Acinetobacter* | 3.00% |
| **6** |  | *Pseudomonas* | 3.79% |  | *Rhodanobacter* | 2.72% |  | *Arthrobacter* | 2.02% |  | *Paenibacillus* | 2.47% |
| **7** |  | *Sphingobacterium* | 3.27% |  | *Chryseobacterium* | 2.39% |  | *Sphingomonas* | 1.99% |  | *Sphingomonas* | 2.24% |
| **8** |  | *Flavobacterium* | 3.19% |  | *Rhizobium* | 2.32% |  | *Chryseobacterium* | 1.59% |  | *Rhizobium* | 1.96% |
| **9** |  | *Janthinobacterium* | 2.66% |  | *Chitinophaga* | 2.06% |  | *Cellvibrio* | 1.57% |  | *Serratia* | 1.89% |
| **10** |  | *Cellvibrio* | 2.58% |  | *Stenotrophomonas* | 1.98% |  | *Rhizobium* | 1.40% |  | *Massilia* | 1.88% |
|  |  |  |  |  |  |  |  |  |  |  |  |  |
| **1** | ZSH | *Sphingomonas* | 7.09% | ZSL | *Sphingomonas* | 7.35% | FSH | *Sphingomonas* | 10.28% | FSL | *Sphingomonas* | 9.89% |
| **2** |  | *Gaiella* | 3.23% |  | *Gemmatimonas* | 3.90% |  | *Gemmatimonas* | 3.63% |  | *Gemmatimonas* | 3.69% |
| **3** |  | *Gemmatimonas* | 3.12% |  | *Gaiella* | 3.04% |  | *Bacillus* | 3.12% |  | *Gaiella* | 2.41% |
| **4** |  | *Bacillus* | 2.72% |  | *Lactococcus* | 2.97% |  | *Gaiella* | 3.04% |  | *Lactococcus* | 2.31% |
| **5** |  | *Saccharibacteria* genera incertae sedis | 2.17% |  | *Bacillus* | 2.29% |  | *Lactococcus* | 2.16% |  | *Bacillus* | 2.13% |
| **6** |  | *Lactococcus* | 2.14% |  | *Spartobacteria* genera incertae sedis | 1.90% |  | *Saccharibacteria* genera incertae sedis | 2.10% |  | *Saccharibacteria* genera incertae sedis | 2.03% |
| **7** |  | *Acidobacteria* Gp3 | 1.94% |  | *Acidobacteria* Gp3 | 1.71% |  | *Acidobacteria* Gp3 | 1.65% |  | *Pseudomonas* | 1.57% |
| **8** |  | *Spartobacteria* genera incertae sedis | 1.76% |  | *Acidobacteria* Gp1 | 1.17% |  | *Spartobacteria* genera incertae sedis | 1.38% |  | *Spartobacteria* genera incertae sedis | 1.31% |
| **9** |  | *Cellvibrio* | 1.52% |  | *Flavobacterium* | 1.13% |  | *Rhodanobacter* | 1.24% |  | *Acidobacteria* Gp6 | 1.18% |
| **10** |  | *Flavobacterium* | 1.51% |  | *Aquicella* | 0.82% |  | *Acidobacteria* Gp6 | 1.17% |  | *Acidobacteria* Gp3 | 1.18% |

**Tab. S4** 18 *Streptomyces* species aligned against the NR database that were possible scab pathogens

| **Species** | **Mean (GSH) ± SD** | **Mean (GSL) ± SD** | **Pct in Bac** | **Pct in Stre** |
| --- | --- | --- | --- | --- |
| *Streptomyces acidiscabies* [1] | 4.78E-03 ± 2.50E-03 | 6.83E-04 ± 3.26E-04 | 0.3365% | 24.6099% |
| *Streptomyces niveiscabiei* [2] | 1.19E-03 ± 5.98E-04 | 2.02E-04 ± 1.05E-04 | 0.0854% | 6.2421% |
| *Streptomyces turgidiscabies* [3] | 5.81E-04 ± 2.79E-04 | 2.06E-04 ± 1.18E-04 | 0.0446% | 3.2599% |
| *Streptomyces scabiei* [4] | 4.12E-04 ± 2.37E-04 | 1.86E-04 ± 1.23E-04 | 0.0301% | 2.2043% |
| *Streptomyces griseus* [5] | 9.46E-05 ± 3.83E-05 | 1.81E-04 ± 8.99E-05 | 0.0114% | 0.8325% |
| *Streptomyces puniciscabiei* [2] | 8.18E-05 ± 3.92E-05 | 8.40E-05 ± 6.64E-05 | 0.0079% | 0.5795% |
| *Streptomyces europaeiscabiei* [6] | 5.96E-05 ± 3.76E-05 | 3.86E-05 ± 3.34E-05 | 0.0046% | 0.3330% |
| *Streptomyces resistomycificus* [7] | 1.38E-05 ± 1.95E-05 | 1.15E-04 ± 8.82E-05 | 0.0041% | 0.2963% |
| *Streptomyces lydicus* [7] | 2.46E-05 ± 2.48E-05 | 7.14E-05 ± 3.44E-05 | 0.0040% | 0.2937% |
| *Streptomyces bottropensis* [8] | 3.74E-05 ± 1.66E-05 | 3.06E-05 ± 1.95E-05 | 0.0034% | 0.2507% |
| *Streptomyces stelliscabiei* [6] | 3.32E-05 ± 1.77E-05 | 1.98E-05 ± 1.25E-05 | 0.0027% | 0.1981% |
| *Streptomyces atroolivaceus* [7] | 5.20E-06 ± 4.97E-06 | 5.68E-05 ± 4.29E-05 | 0.0020% | 0.1450% |
| *Streptomyces aureofaciens* [5] | 1.16E-05 ± 5.41E-06 | 2.54E-05 ± 2.50E-05 | 0.0015% | 0.1112% |
| *Streptomyces ipomoeae* [9] | 2.40E-05 ± 3.44E-05 | 1.32E-05 ± 1.12E-05 | 0.0014% | 0.1060% |
| *Streptomyces luridiscabiei* [2] | 7.80E-06 ± 1.37E-05 | 1.08E-05 ± 1.00E-05 | 0.0010% | 0.0748% |
| *Streptomyces diastatochromogenes* [7] | 2.40E-06 ± 2.30E-06 | 1.42E-05 ± 1.65E-05 | 0.0006% | 0.0455% |
| *Streptomyces corchorusii* [7] | 2.20E-06 ± 4.38E-06 | 2.00E-07 ± 4.47E-07 | 0.0002% | 0.0137% |
| *Streptomyces rochei* [10] | 2.00E-07 ± 4.47E-07 | 2.80E-06 ± 3.03E-06 | 0.0001% | 0.0078% |
| sum | 7.37E-03 ± 3.61E-03 | 1.94E-03 ± 9.30E-04 | 0.5416% | 39.6040% |

Pct in Bac: the percentage of a taxon in bacteria; Pct in Strep: the percentage of a taxon in the genus *Streptomyces*

**Tab. S5** Strains isolated from the culture experiment with the phylogenetic similarity to pathogenic *Streptomyces*

| Isolated strain | Hit strain | | |
| --- | --- | --- | --- |
|  | Strain name | Sequence ID | Identity |
| FD1-9 | *Streptomyces acidiscabies* strain ATCC 49003 | NR_116534.1 | 99.84% |
| OD3-9 | *Streptomyces acidiscabies* strain ATCC 49003 | NR_116534.1 | 99.84% |
| OH1-5 | *Streptomyces acidiscabies* strain ATCC 49003 | NR_116534.1 | 99.84% |
| OH1-10 | *Streptomyces acidiscabies* strain ATCC 49003 | NR_116534.1 | 99.84% |
| PH3-10 | *Streptomyces acidiscabies* strain ATCC 49003 | NR_116534.1 | 99.84% |
| YD1-1 | *Streptomyces acidiscabies* strain ATCC 49003 | NR_116534.1 | 99.84% |
| YD1-11 | *Streptomyces turgidiscabies* strain ATCC 700248 | NR_040828.2 | 99.92% |
| YD1-14 | *Streptomyces acidiscabies* strain ATCC 49003 | NR_116534.1 | 99.84% |
| YD4-1 | *Streptomyces acidiscabies* strain ATCC 49003 | NR_116534.1 | 99.84% |
| YH1-1 | *Streptomyces acidiscabies* strain ATCC 49003 | NR_116534.1 | 99.84% |
| YH1-6 | *Streptomyces acidiscabies* strain ATCC 49003 | NR_116534.1 | 99.84% |
| YH3-1 | *Streptomyces acidiscabies* strain ATCC 49003 | NR_116534.1 | 99.84% |

**Supplementary References**

1. Lambert DH, Loria R. *Streptomyces acidiscabies* sp. nov. International Journal of Systematic and Evolutionary Microbiology. 1989;39(4):393-6.
2. Park DH, Kim JS, Kwon SW, Wilson C, Yu YM, Hur JH, et al. *Streptomyces luridiscabiei* sp. nov., *Streptomyces puniciscabiei* sp. nov. and *Streptomyces niveiscabiei* sp. nov., which cause potato common scab disease in Korea. International Journal of Systematic and Evolutionary Microbiology. 2003;53(6):2049-54.
3. Miyajima K, Tanaka F, Takeuchi T, Kuninaga S. *Streptomyces turgidiscabies* sp. nov. International Journal of Systematic and Evolutionary Microbiology. 1998;48(2):495-502.
4. Lambert DH, Loria R. *Streptomyces scabies* sp. nov., nom. rev. International Journal of Systematic and Evolutionary Microbiology. 1989;39(4):387-92.
5. Corbaz R. Etude des streptomycetes provoquant la gale commune de la pomme de terre. Journal of Phytopathology. 1964;51(4):351-60.
6. Bouchek-Mechiche K, Gardan L, Normand P, Jouan B. DNA relatedness among strains of *Streptomyces* pathogenic to potato in France: description of three new species, *S. europaeiscabiei* sp. nov. and *S. stelliscabiei* sp. nov. associated with common scab, and *S. reticuliscabiei* sp. nov. associated with netted scab. International journal of systematic and evolutionary microbiology. 2000;50(1):91-9.
7. Archuleta JG, Easton GD. The cause of deep-pitted scab of potatoes. American Potato Journal. 1981;58(8):385-92.
8. Zhou B, Zhang MS, Ma XK. First report of *Streptomyces bottropensis* causing potato common scab in Hebei Province, China. Plant Disease. 2017;101(3):502.
9. Clark CA, Chen C, Ward-Rainey N, Pettis GS. Diversity within *Streptomyces ipomoeae* based on inhibitory interactions, rep-PCR, and plasmid profiles. Phytopathology. 1998;88(11):1179-86.
10. Bramwell PA, Wiener P, Akkermans ADL, Wellington EMH. Phenotypic, genotypic and pathogenic variation among streptomycetes implicated in common scab disease. Letters in applied microbiology. 1998;27(5):255-60.
